# Nano- and Microemulsions in Biomedicine: From Theory to Practice

**DOI:** 10.3390/pharmaceutics15071989

**Published:** 2023-07-20

**Authors:** Boris Nikolaev, Ludmila Yakovleva, Viacheslav Fedorov, Hanmei Li, Huile Gao, Maxim Shevtsov

**Affiliations:** 1 Laboratory of Biomedical Nanotechnologies, Institute of Cytology of the Russian Academy of Sciences (RAS), Tikhoretsky Ave. 4, 194064 Saint Petersburg, Russia; nikolaevhpb@gmail.com (B.N.); yakluda5@gmail.com (L.Y.); fedorovvs.biotech@gmail.com (V.F.); 2 Personalized Medicine Centre, Almazov National Medical Research Centre, Akkuratova Str. 2, 197341 Saint Petersburg, Russia; 3 Department of Inorganic Chemistry and Biophysics, Saint-Petersburg State University of Veterinary Medicine, Chernigovskaya Str. 5, 196084 Saint Petersburg, Russia; 4 School of Food and Biological Engineering, Chengdu University, Chengdu 610106, China; lihanmei@cdu.edu.cn; 5 Key Laboratory of Drug-Targeting and Drug Delivery System of the Education Ministry, Sichuan Engineering Laboratory for Plant-Sourced Drug and Sichuan Research Center for Drug Precision Industrial Technology, West China School of Pharmacy, Sichuan University, Chengdu 610064, China; gaohuile@scu.edu.cn; 6 Department of Radiation Oncology, Technical University of Munich (TUM), Klinikum Rechts der Isar, Ismaninger Str. 22, 81675 Munich, Germany; 7 Laboratory of Biomedical Cell Technologies, Far Eastern Federal University, 690091 Vladivostok, Russia

**Keywords:** nanoemulsions, microemulsions, burn treatment, controlled release, wound healing, cancer diagnostics, drug delivery

## Abstract

Nano- and microemulsions are colloidal systems that are widely used in various fields of biomedicine, including wound and burn healing, cosmetology, the development of antibacterial and antiviral drugs, oncology, etc. The stability of these systems is governed by the balance of molecular interactions between nanodomains. Microemulsions as a colloidal form play a special important role in stability. The microemulsion is the thermodynamically stable phase from oil, water, surfactant and co-surfactant which forms the surface of drops with very small surface energy. The last phenomena determines the shortage time of all fluid dispersions including nanoemulsions and emulgels. This review examines the theory and main methods of obtaining nano- and microemulsions, particularly focusing on the structure of microemulsions and methods for emulsion analysis. Additionally, we have analyzed the main preclinical and clinical studies in the field of wound healing and the use of emulsions in cancer therapy, emphasizing the prospects for further developments in this area.

## 1. Introduction

It is difficult to name the areas of science and technology in which emulsions would not be used. The physical phenomena that determine the stability of emulsions and their structure are subject to general laws that have found detailed coverage in the relevant sections of colloidal chemistry. At the same time, the use of emulsions in medicine, technical microbiology, engineering enzymology and other areas of modern biotechnology have their own characteristics. The fine structural organization of protein molecules and other more complex biological formations, which determines their lability, requires “soft” technological influences. In this regard, the success of the final result depends primarily on the chemistry of the strong nature of the components of the emulsion systems and methods for their preparation. There is also an increase in scientific interest in microheterogeneous systems with a limiting degree of emulsification—the so-called microemulsions. According to the generally accepted point of view, the term microemulsion originates from the thermodynamically stable, isotropic phase in the form of a fine dispersion consisting of two immiscible liquids. The fine dispersion from water and oil is stabilized with help of surfactant and co-surfactant compounds. The microemulsion is considered to comprise an ensemble of nanodomains with sizes much less than the wavelength of visible light. The size of structural element of microemulsion (water drop or nanodomain of bicontinuous colloid) has a small dimension in the range of 10–100 nm. The last consideration leads to a confusion of definitions relative to nanoemulsions and microemulsion based on criterion of rate dispersion. With regards to nanoemulsions, these dispersions have a lifetime finality owing to non-favorable energy and entropy contributions to the mixture. It should be noted that the microemulsion phase can fulfil the role of a stabilizing factor for nano- and macroemulsions by formation of an interfacial layer around fluid particles. Therefore, special attention is paid to the study and theoretical description of the structure of microemulsion systems and questions of their practical application.

In the proposed work, an attempt was made to present a number of the most typical areas of biotechnology, in which interfacial phenomena and the dispersed nature of a biological preparation are essential for its production and use. The main types of emulsions, the characteristics of stabilizers, and the physicochemical behaviors of multicomponent systems of various dispersions used in biotechnological processes are described. Particular attention is paid to inverse emulsions. The choice of factual material is related to fields such as biotechnology related to the production of biologically active microcapsules, microspheres, emulsion forms of entomopathogenic drugs, prolonged dosage forms and diagnostic tools. Emulsified systems have found their use in medicine. For example, the skin is a remarkable organ, comprising 1/10 of the body’s weight, and acting as a base location for drug administration. However, it is also a highly efficient barrier, meaning that conventional transdermal compositions will have lower biodistribution. In this case, microemulsions have several great advantages: aside from requiring little to no energy to form, they are notably stable during storage, with some forms possessing the ability to re-emulsify. The low viscosity of microemulsions and low droplet size allow for better drug delivery, where these robust systems are able to solve hydrophilic and lipophilic therapeutic agents, as well as incapsulate insoluble pharmaceuticals. The same applies to oral, nasal or targeted delivery. Microemulsions are able to absorb large amounts of lipophilic substances, easily penetrate into skin and are bioavailable as carriers for proteins. Our previous studies have shown the possible coexistence of nanoclusters of bactericide Ag with immunoactive proteins in the microemulsion phase. Such colloidal systems are convenient for the treatment of trophic ulcers and burns. The surfactant coat on nanodrops protect the biolabile molecules from the impact of chemically active Ag. The parenteral microemulsion application of pharmaceuticals has no disadvantage of probable thrombosis. Microemulsion formulations with absorbed enzymes (e.g., lipases) have also found their niche in therapy. Enzymatic catalysis in microemulsions is realized in a number of biochemical reactions of peptides and sugars. Microemulsion technology is successfully developed for the delivery of poorly soluble drugs. Self-emulsifying drug delivery systems are recommended for enhanced administration to disease organs by oral, parenteral and topic methods. Thus, the versatile properties of the microemulsion state are the promising and interesting subject for development of new effective pharmaceutical delivery means.

## 2. Theoretical Basics for Emulsions

According to the generally accepted definition, emulsions are dispersed systems in which one liquid macrophase is distributed inside of another. In the classical case, emulsions are formed from mutually insoluble liquids, one of which is usually a polar solvent, like water, and the other is a hydrophobic solvent, more commonly called oil for short. Depending on which phase is continuous, emulsions are divided into direct emulsions, where the hydrophobic solvent is dispersed in water (o/w), and reverse (w/o), in which water particles are distributed in oil [1,2]. With double emulsification, multiple emulsions can be obtained in which the dispersed phase, being microheterogeneous, combinations of w/o and o/w dispersions. Such emulsions often form in the vicinity of the phase reversal point. Multiple emulsions can be roughly divided into w/o/w and w/w/o systems.

As is known, all macroscopic emulsions are thermodynamically irreversible systems; nevertheless, their kinetic stability can vary over a wide range. For example, some emulsions do not separate for several years. In practice, the stability of emulsions is ensured by introducing various types of emulsifiers into dispersions, which, being adsorbed in the surface layer, reduce tension or form a structural–mechanical barrier that prevents drops from approaching each other.

**Figure 1:**
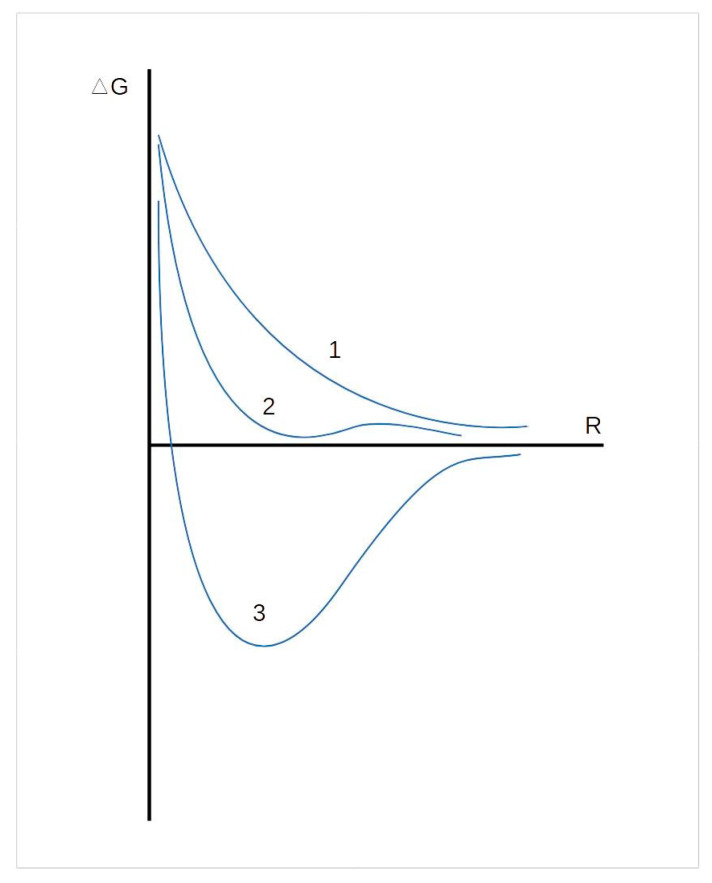
Potential energy isotherms depending on droplet radius. 1—no interaction, 2—weak interaction, 3—strong interaction.

Wide application in biotechnology is found for emulsions of the w/o type. The kinetic stability of dilute inverse emulsions is proportional to the viscosity of the continuous medium and inversely proportional to the square of the particle radius, and, in the case of concentrated systems, is small. Aggregative stability of inverse emulsions, i.e., the ability to resist coagulation is determined by the total interaction energy of drops, which is the sum of the repulsion energy of double electric layers and the van der Waals attraction of drops. Depending on the type of the emulsions under consideration, several types of isotherms of the potential energy of interaction between drops can be expected, reflected in potential energy (Figure 1).

The DLVO theory describes in general terms the patterns of coagulation of inverse emulsions [3] without involving the characteristics of the structure of the interfacial layer, the value of which increases sharply when coalescence processes are considered. Coalescence is the process of merging drops with the release of a substance in the form of a separate homogeneous phase. The stability of inverse emulsions with respect to coalescence depends to a greater extent on the structure of the interfacial layer formed by surfactant molecules, and not on the component composition of water droplets. It is this circumstance that makes it possible to obtain stable emulsions containing substances of various nature in the dispersed phase. The coalescence of water droplets in inverted emulsions largely depends on the disjoining pressure *P*(*h*) that occurs in a thin film when the particles approach each other. By definition, the disjoining pressure *P*(*h*) is equal to the difference between the normal component *P_N_* of the pressure tensor in the interlayer and the pressure *P*_0_ in the phase from which it was formed as a result of thinning [3,4]:

(1) $$P\left(h\right)=P_{N}-P_{0}={\left(\frac{\partial G}{\partial h}\right)}_{T,P,\mu }\;$$

where *G* is the Gibbs free energy, *µ* is the chemical potential, *T* is the temperature, *P*_0_ is the pressure and *h* is the distance between drops.

The disjoining pressure *P*(*h*) in the hydrocarbon film between two layers of the aqueous phase can be represented as the sum

(2) $$P\left(h\right)=P_{v}+P_{el}+P_{ads}$$

where *P_v_*, *P_el_* and *P_ads_* are the van der Waals, electrostatic and adsorption components.

In oil films in an aqueous medium, the van der Waals interaction leads to a negative component of the disjoining pressure *P_v_*. For the formation of stable inverse emulsions, it is necessary to have *P* > 0, ∂*P*/∂*h* < 0. The nature of the positive component *P*(*h*) of thin films has not been finally established. It is assumed that the main role in increasing the stability of inverse emulsions belongs to adsorption and structural–mechanical barriers [3,4,5].

The choice of emulsifiers to stabilize emulsions is carried out using the semi-empirical scales of hydrophilic–lipophilic balance (HLB) group numbers. Table 1 shows the effect of surfactant type, as determined by the characteristic number of HLB, on the type of emulsion formed. By selecting a surfactant with the required number of HLBs, it is possible, in principle, to create emulsions of both direct and reverse types. To stabilize multiple emulsions, a combination of phases of two emulsifiers with different HLBs indices is usually used. However, it should be remembered that such recommendations are approximate, since the conditions for the formation of an emulsion and the specific chemical structure of the surfactant molecule sometimes make their own adjustments.

The advantage of the HLB scale is that it allows for the targeted selection of emulsifiers and the calculation of HLB numbers using the amphiphilic compound formula based on the principle of group number additivity. Repeated attempts have been made to fill the empirical Griffin scale with physical content. Additionally, surface properties for surfactants were described in [6]. Without dwelling on a detailed review of all the numerous attempts to interpret the HLB number scale, we note the work of A.I. Rusanov and V.L. Kuzmin [7], where ideas about the paramount importance of the work of adsorption of surfactant molecules during the transfer of a substance from oil β to water α are developed. The work of transfer of a single molecule Wαβ is equal to:

(3) $$W^{\alpha \beta }=k*T*\ln\left(\frac{C^{\alpha }}{C^{\beta }}\right)$$

where *C*^α^ and *C*^β^ are the component concentrations for each phase.

Based on Davis’ *HLB* equation:

(4) $$HLB=7+0.36*\ln\left(\frac{C^{\alpha }}{C^{\beta }}\right);$$

it may be written as:

(5) $$HLB=7+0.36*\;W^{\alpha \beta }/kT,$$

which is the thermodynamic definition of the *HLB* scale. The first member of the expression is chosen for reasons of convenience in operating with positive numbers. Definition (5) satisfies the criteria of group number additivity and is also suitable for micelle-forming surfactants.

Guided by the above provisions of the theory, it is possible to formulate some criteria for choosing an emulsifier and component composition when obtaining an emulsion:

1. The surfactant used should prevent the rupture of thin films, i.e., be a barrier that prevents particles from approaching at a distance less than twice the length of the surfactant molecule;
2. The “head” of a surfactant molecule, which is in the continuous phase, must be larger than the diameter of the “tail” of the molecule;
3. The surfactant should dissolve better in the continuous phase;
4. The adsorbed interfacial surfactant layer must have sufficient strength, i.e., be characterized by a large value of the surfactant desorption work;
5. The formation of a structural–mechanical barrier of increased viscosity contributes to the stabilization of reverse emulsions;
6. The protective adsorption layer is formed at the maximum saturation of the surface (Γ → Γ_∞_) of the interfacial layer.

Correlating these recommendations with experience in the formation of inverse emulsions w/o with biotechnological fillers, we can state the following: stable inverse emulsions are formed when using detergents with HLB = 1–10. Optimum dispersion down to 0.1 µm is achieved in the transition region close to the phase inversion boundary. As surfactants, oil-soluble non-ionic detergents of the polyoxyethylated series are more preferred. The range of surfactants is quite wide, covering the scale of HLB numbers from 1 to 40. Most known emulsifiers are either salts of fatty acids and amines, or ethoxylated adducts of hydrophobic molecules (Table 2). Depending on the number of hydrophilic hydroxyethyl groups and hydrophobic –CH_2_ groups of the aliphatic part of the surfactant molecule, the HLB index changes by tens of units.

The features of surfactants and the areas of their application in the national economy are described in detail in the literature [5,6,7,8]. In addition to chemical methods for the production of emulsifiers, there are biological methods for the production of surfactants. One of the promising sources of emulsifiers are microorganisms. Amphiphilic molecular formations are part of the cell membranes of microorganisms in the form of lipids, glycolipids and lipopeptides. These biosurfactants of microbial origin are used in the pharmaceutical industry, in the processes of secondary oil refining and biotechnology.

Due to the prevalence and wide variety of ethoxylated surfactants, these products occupy the main place among non-ionic emulsifiers with a biologically mild effect. In a detailed review [8] of a large amount of experimental material, the scope of applications of ethoxylated surfactants in chemical technology is disclosed. Using the example of ethoxylated paraffin adducts, the main features of the structure of inverse emulsions in the homologous series of this class of detergents are considered. The structural features of such emulsions are that micron-sized water droplets (emulsified water) and nanometer-sized water microdroplets (microemulsified water) coexist in the dispersed phase. The stability of such emulsions is provided by a hydrophobic layer consisting of a microemulsion phase.

When creating inverse emulsions, carriers of biologically active compounds and whole cells, it is necessary to take into account the localization of the active principle in the drop and its effect on the surface tension γ between water and oil. This circumstance is essential in obtaining various emulsion forms of biological preparations. For example, E. coli cells, which have a hydrophilic surface, are localized inside droplets without contact with the oil phase. The bacterial cells of C. Guilliermondii, C. maltosa, and C. sake, which consume normal paraffins as a carbon source, have hydrophobic surface areas and, therefore, are adsorbed at the w/o interface during emulsification. [9,10].

## 3. Emulsification Strategies in Biotechnology

The physicochemical properties of emulsions depend not only on the component composition of the systems, but also on the methods of their preparation. For the preparation of macroemulsions, typical emulsification methods with a low energy consumption for droplet separation in a local zone are suitable. During the preparation of the o/w emulsion, the oil phase is added in portions to water, with the emulsifier being dissolved in one of the phases before mixing, depending on the type of surfactant selected on the HLB scale. w/o type emulsions are usually prepared by introducing aqueous systems into an oil solution of an emulsifier while stirring the emulsion. Stable double emulsions are usually prepared by re-emulsifying w/o or o/w type primary emulsions in water or oil, respectively.

In industry and laboratory practice, the mixing of phases is carried out in apparatuses with mixers of various types, in which a continuous or fractional supply of the emulsifiable mixture is carried out into the continuous phase. The effectiveness of emulsification depends on the time and intensity of mixing, and emulsification parameters are optimized empirically, since they depend on the type of device and the nature of the emulsifier. Emulsions obtained in this way are distinguished by a noticeable polydispersity, and their storage and use is limited due to the low kinetic stability of the dispersion. Therefore, in the food and pharmaceutical industries, homogenization is often used, i.e., secondary preparation of the emulsion, aimed at reducing the size of the droplets of the dispersed phase. Homogenization is carried out by forcing the initial emulsions through filters with small holes under high pressure. Homogenized emulsions are more stable than the original emulsions and can be stored without separation for several months. Various types of homogenizers differ in performance related to the size of the holes, the total area of the dies and the range of operating pressures.

Stable emulsions with a narrow distribution of droplets of the dispersed phase are obtained by irradiation with ultrasound. The use of this dispersion technology, however, has a number of limitations, since cavitational effects can change the properties of some emulsifiers, as well as labile biological objects. Jet generators or liquid whistles are widely used in emulsification technology [11,12]. The principle of operation of the jet generator is quite simple and effective. The mixture to be emulsified is pumped under pressure at a speed of up to 50 m/s through a converging nozzle onto a thin elastic plate in which sound vibrations are excited. By selecting the geometric dimensions of the plate, the pressure and the distance between the nozzle and the plate, the resonance frequency of the plate oscillations is achieved, which is most often 25–30 kHz. Jet generators make it possible to obtain emulsions with a droplet size not lower than those obtained in homogenizers, but with greater productivity and energy efficiency for increasing interfacial energy.

The choice of emulsification method sometimes affects the type of emulsion formed. With intensive stirring, it is sometimes possible to obtain o/w tin emulsions with an excess of the hydrophobic phase. Such kinetically unstable emulsions can undergo phase reversal with a slight change in temperature, the addition of another surfactant, or contact with a foreign surface of the vessel. The phenomenon of phase reversal can be used to increase the dispersion of the system, because it was noticed that when passing the point of inversion, additional fragmentation of the drops occurs. In laboratory practice, the method of emulsification according to Kremlev, as described in [13], has found application.

The listed examples of dispersion give emulsions with a droplet size of the dispersed phase from 0.1 μm to 100 μm. The desire to obtain highly dispersed emulsions by increasing the intensity and duration of emulsification encounters fundamental difficulties associated with an increase in the interfacial energy of droplets during crushing. Upon reaching a certain critical radius RC (0.1 cm), the coalescence of droplets begins to compete with the process of destruction and further dispersion becomes impossible [14]. Attempts to improve the design of dispersants, the search for new methods of processing dispersions with ultrasound, shock effects of temperature and pressure do not significantly improve the dispersion of the emulsion.

According to the general provisions of the theory, an increase in the degree of dispersion of microdroplets can be achieved by lowering the interfacial tension at the water–oil interface. As Shulman’s classical work [15] showed, formation of a surface layer of droplets with the help of well-balanced amphiphilic molecules makes it possible to drastically reduce the energy costs for the formation of a developed emulsion surface. Moreover, in some systems such low values of surface energy are achieved when the so-called self-emulsification phenomenon occurs. Moreover, the introduction of aliphatic alcohols as a codetergent into the surface layers of emulsions in some cases gives microheterogeneous thermodynamically stable systems. Such systems, called microemulsions, do not require special methods and devices for emulsification and almost immediately after mixing form colloidal dispersions with a particle size of up to several tens of nanometers. According to the type of dispersed phase, microemulsions are divided into direct (o/w) and reverse (w/o), the latter have found application in biotechnology.

Additionally, bicontinuous microemulsions are an example of coexisting interconnected water and oil droplets, spontaneously formed upon phases being covered with one or multiple surfactant films at low concentrations. Both water and oil there do not form independent droplets, existing rather in the form of disordered nanoscale domains. These structures are characterized by high conductivity and diffusion, and have found use as a controlled media for nanoscale synthesis [16].

**Figure 2:**
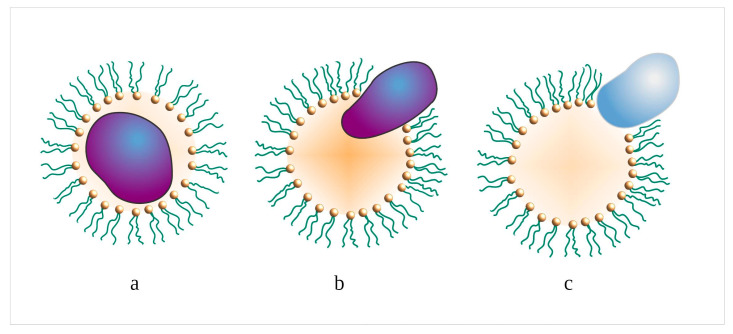
Schematics for macromolecule embedding: (**a**) lipophilic, (**b**) amphiphilic, (**c**) hydrophilic.

Various biologically active substances, such as proteins, antibiotics, amino acids, etc., can be included in the dispersed phase of microemulsions by the solubilization mechanism. Depending on the type of protein, several variants for embedding the molecule into macrodroplets are possible, as shown in Figure 2. Hydrophilic proteins, as a rule, are localized in the water core, amphiphilic macromolecules partially penetrate deep into the surface layer, and hydrophobic proteins come into contact with an organic solvent. Obviously, in microemulsions of the o/w type, the nature of the distribution of macromolecules between the continuous phase and the dispersion medium differs from the previous case only in that the hydrophilic proteins are in the continuous phase.

There are several methods for introducing proteins into reverse microemulsions: solubilization of small volumes of an aqueous protein solution with a detergent solution in an organic solvent, dissolution of a lyophilized protein in surfactant solutions with a given hydration number, and protein extraction in a two-phase system consisting of comparable volumes of an aqueous protein solution and a hydrophobic solvent with a surfactant. At present, the preservation of the enzymatic activity of many biocatalysts in microemulsions of the w/o type [16,17] has been reliably established, which, being the subject of research in the so-called micellar enzymology, has undoubted practical significance.

At present, microemulsions are being intensively studied from the point of view of condensed-state physics. It turned out that the structure of microemulsions is extremely labile and diverse. The structural features of microemulsions are much more complex and diverse than macroemulsions. As noted earlier, in w/o-type macroemulsions stabilized with ethoxylated surfactants, there is a microemulsion phase, which has a significant effect on the stability of inverse emulsions. Considering the wide possibilities of using such systems in biotechnology, in the next section we will consider the features of the structural organization and structure of microemulsions in the light of modern theoretical concepts.

## 4. The Structure of Microemulsions and Methods of Its Investigation

The physicochemical properties of emulsions depend not only on the component composition of the systems, but also on the methods of their preparation. For the preparation of macroemulsions, typical emulsification methods with a low energy consumption for droplet separation in a local zone are suitable. 

In 1943, Shulman [15] made an interesting observation of the phenomenon of the complete clearing of an oil-in-water emulsion after the addition of aliphatic alcohols. Schulman called these four-component systems microemulsions (MEs). Subsequently, other new compositions were obtained from water, oil, surfactants and alcohols with similar properties. In contrast to micellar solutions, water and the hydrophobic component are included in microemulsions in comparable amounts. A distinctive feature of MEs is their extremely high stability. For several years of storage, MEs do not change their properties at all. The latter prompted researchers to think about their thermodynamic stability, which, in principle, makes them a new class of colloidal systems. Despite the great work done in the field of structural analysis of MEs, there is still no generally accepted definition of microemulsions, which is associated with a variety of properties exhibited by varying the composition and temperature in pouring experiments. Currently, the definition of a microemulsion as a thermodynamically stable isotropic microheterogeneous lyophilic system created by mixing comparable amounts of water or aqueous solutions of electrolytes, a hydrophobic solvent (oil), surfactant and codetergents (alcohols) is distinguished by the greatest generality. Some authors consider it necessary to add the presence of opalescence of solutions to the listed features as an essential feature. It is obvious that this definition is also not sufficiently rigorous and complete, since it does not clearly emphasize the difference between ME and micellar solutions. Recently, microemulsions have been obtained in which water is replaced by other polar solvents—glycerol, polyethylene glycol, etc.—which once again indicates the need for a broader interpretation of the above definition. In the following discussion, the totality of the main characteristics of these systems is analyzed.

**Figure 3:**
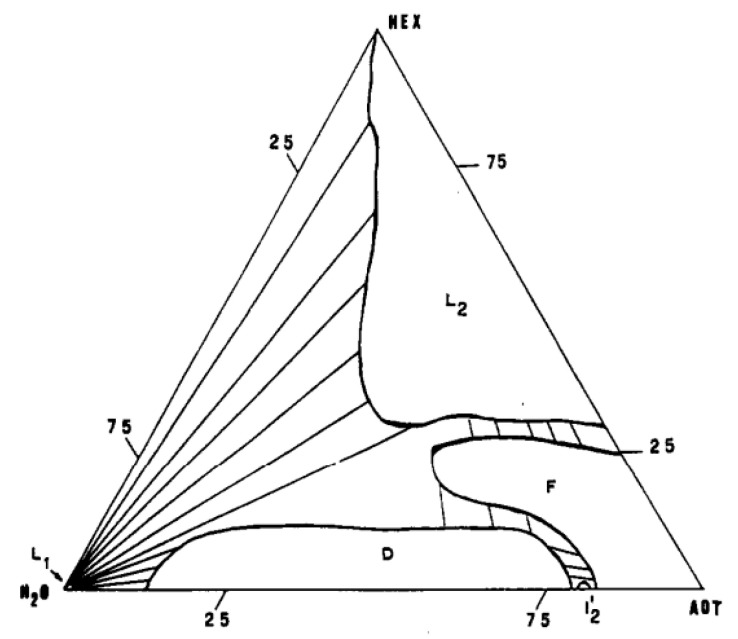
Phase diagram of the water–hexane–AOT system at 20 °C. The phase denomination is as follows: Li, solution phase; L2, reverse solution phase; D, lamellar phase; I/, viscous isotropic phase; F, reverse hexagonal phase [16].

The regions occupied by microemulsions as thermodynamically stable phases represent vast zones on phase diagrams [18]. In this regard, the phase diagram of the water–oil–AOT (sodium salt of diethylhexylsulfosuccinic acid) system (Figure 3) is indicative, which is currently the most thoroughly studied object of the microemulsion state. The water content in the region of a single-phase state can vary over a wide range. The microemulsion region is adjacent to the regions of multiphase equilibria. In the upper corner of the diagram, the microemulsion zone smoothly passes into the region of inverted aqueous micelles in a hydrophobic solvent. Along the delamination boundary, the microemulsion passes into the emulsion state. Within region I, the ME structure varies depending on the composition.

The use of various methods of structural analysis makes it possible to establish the type of dominant colloidal structures. According to the type of structural element, MEs are divided into several types, described by the following models: [15,19,20].

1. Drip or micellar model;
2. Bicontinuous models;
3. Talmon-Prager–De Gennes model;
4. Critical state model.

The complexity of the ME structure is such that it is often necessary to use several model approaches to describe a monovarious region. The development of model ideas about ME is of great importance for understanding the mechanism of ME stability and is still far from complete.

According to the ME droplet model, which logically follows from macroemulsion views, a dispersed ME particle is a drop of water or oil coated with a monomolecular layer of surfactant. ME stabilization is achieved by lowering the surface tension of the drop *γ* according to the formula:

(6) γ=γow−π

where *π* is the surface tension and *γ_o/w_* is the oil/water ratio.

Taking the drop radius as equal to 10 nm, according to theoretical estimates, one should expect a decrease in the surface tension of the film to 10 dynes/cm, but the experimental values of *γ* turn out to be even less by three orders of magnitude. This fact cannot be satisfactorily explained within the simplified droplet model. At the same time, advantages of the model such as clarity in operating with the concept of film interfacial tension has stimulated attempts to further develop this model by taking into account the entropy contribution of the random distribution of drops in the volume. Thus, the reduction in the free energy of the dispersion ∆G is considered as the sum:

∆G = ∆G_1_ + ∆G_2_ + ∆G_3_ (7)

where ∆G_1_ is the free energy for interphase, ∆G_2_ is the free energy for droplet collision and ∆G_3_ is the entropic configuration.

The criteria for droplet stability in dispersion are:

(8) $$\frac{\partial G}{\partial R}{|}_{R=R_{0}}=0,\;\;\frac{\partial^{2}G}{\partial^{2}R}{|}_{R=R_{0}}=0$$

Calculating ∆G_2_ according to the DLVO theory, and the entropy contribution according to random statistics, several types of potential functions are obtained, some of which have a region of thermodynamic stability. Specific calculations of ∆G are extremely laborious due to the need to take into account the potential of the ionic atmosphere of the Gouy–Chapman–Stern layer and van der Waals interactions between drops [21]. The drip model is most realistic near the vertices of phase diagram triangles, i.e., when there is an excess of water or oil in the solution. The droplet model was confirmed by small-angle X-ray scattering of light and neutrons for microemulsions formed by AOT and sodium dodecyl sulfate. In the case of non-ionic surfactants, the structure of the microemulsion is more complex, although even here, with an excess of water or oil, data were obtained on the existence of contrast-pronounced particles with a monomolecular surfactant film.

**Figure 4:**
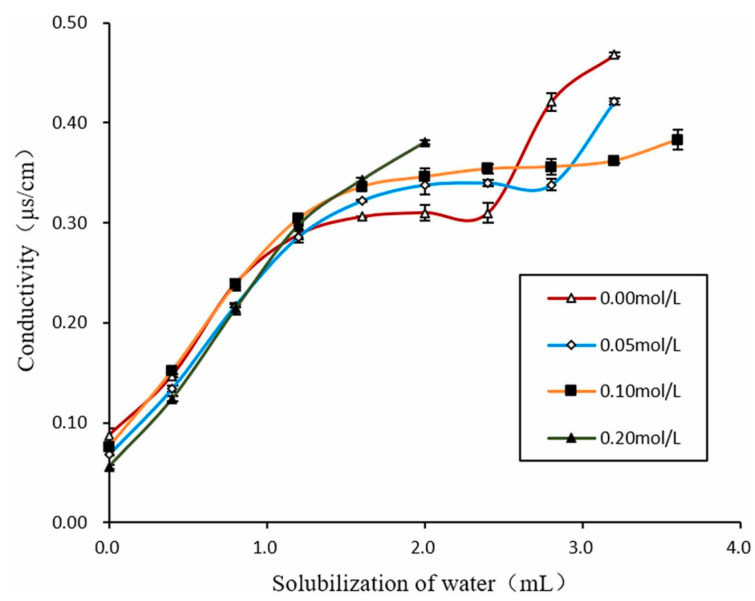
Changes in conductivity of AOT microemulsion system with water addition at different KCl concentrations [23].

The drop model of the structure of microemulsions is a kind of pseudo-phase approximation, which assumes the possibility of considering the interfacial film as a separate phase. The added codetergents are also completely localized in the surface layers of herons. The droplet approximation assumes the isolation and weakness of the interaction between particles. With an increase in the number of particles in the volume (this case is most interesting, since high solubilization of water-soluble biological substrates is achieved in this case), interparticle interactions increase, leading to droplet deformation. At comparable concentrations of water and oil in the diagram in the region of a single-phase state, one should expect a sharp structural transition of w/o droplets into a dispersion of w/w droplets, similarly to the usual inversion of emulsions. However, this does not happen. Measurements of electrical conductivity in most MEs do not detect a jump in conductivity [22]. As can be seen from the curve of the concentration behavior of the water–isooctane–AOT microemulsion, the conductivity changes smoothly from high to low with added electrolytes (Figure 4). 

The continuous nature of the curves in the region of possible inversion is explained by the percolation mechanism, which is inconsistent with the “droplet” concept of the dispersion of isolated spheres. To resolve this contradiction, the theory followed the path of creating bicontinuum models of mutually penetrating spheres (networks) of water and a hydrophobic solvent with a fluctuating structure. A fluctuation structure similar to that shown in Figure 3 ensures a charge transfer through the percolation mechanism. This model assumes the existence of an easily bent interface with large-scale fluctuations. De Gennes proposed introducing the correlation length parameter ξ to describe such a surface, defining it as the distance at which the surface orientation does not change noticeably [24]. Pieces of the interfacial surface of area ξ2 have an independent random orientation and break up the volume of oil and water into parts of indeterminate forts connected to each other. A fluctuating surface can make a significant entropy contribution to the free energy of microemulsions. With a high content of surfactants in the microemulsion, the surface rigidity can increase to the level of rigidity of liquid crystal structures (domains). For example, AOT, when mixed with water, gives liquid crystals of the lamellar and hexagenal type, which, upon oil solubilization, are destroyed, passing into low-viscosity isotropic microemulsion states. To describe the statistics of random orientation of the interfacial surface, either regular Talmon-Prager models [25,26] or Voronoi models are used.

The surface energy of the ME interfacial layer γξ^2^ is comparable with the Boltzmann characteristic of thermal motion kT [25]. Talmon-Prager related the entropy of the ME system to the surfactant concentration [D] under the assumption of a constant cross section of the polar “head” of the molecule and obtained the expression:

(9) $$S=p{\left[\frac{\left[D\right]}{\varphi \left(1-\varphi \right)}\right]}^{3}\left[3ln\frac{\left[D\right]}{\left(1-\varphi \right)}+\varphi ln\varphi +\left(1-\varphi \right)\ln\left(1-\varphi \right)\right]$$

where *p* is constant and *φ* is the volumetric measure of oil content.

At temperatures close to the ME phase separation points, De Genne’s formalism merges with theories of the critical state of matter, in which the parameter ξ becomes equal to the correlation radius of concentration fluctuations [27]. The degree of severity of the critical transition varies depending on the composition and type of ME. Critical phenomena during separation are found both in binary solutions of ethoxylated ethers and in MEs based on them.

Our knowledge about the structure of a microemulsion, due to the variety of manifested physical effects of a fundamental property (the behavior of an ensemble of a countable number of particles, the special properties of water, the critical state), is determined by physical methods for obtaining structural information. The realism of model ME patterns largely depends on the correct interpretation of the chosen study parameters and the physical method itself. The high dispersity of the ME, at which the size of the structural element is comparable to the wavelength of light, causes strong Rayleigh scattering. The static light scattering method was used to measure the characteristic particle sizes of microemulsions produced by solutions of surfactants of various nature. The intensity of light of a certain wavelength λ, scattered by the sample at an angle θ, is determined by the formula:

(10) $$J\left(\bar{q},\varphi \right)=K\varphi S\left(q,\varphi \right)P\left(q\right)$$

where *K* is constant, $\bar{q}$ = 2π/λsinθ is the wave vector and *φ* is the particle volume.

For spherical particles with R radius:

(11) $$P\left(q\right)={\left\{3\sin\left(\bar{q},\;r\right)-\bar{q},\;rcos\frac{\left(\bar{q},\;\;r\right)}{{\left(q,r\right)}^{3}}\right\}}^{2}$$

Structural factor *S*(*q*) may be evaluated, if we use the laws for particle interaction. *S*(*q*) is measured by a radial function for particle displacement *q*(*r*) in this way:

(12) $$S\left(q\right)=1+\frac{4\pi N}{q}\overset{\infty }{\underset{0}{\int }}\left[q\left(r\right)-1\right]rsin\left(q,r\right)dr$$

where *N* is the number of particles.

If *φ*, *q* → 0 the equation transforms, causing:

(13) $$S_{\varphi \to 0}\left(q,0\right)=1$$

(14) $$S_{q\to 0}\left(q,\varphi \right)=\frac{KT}{V}{\left(\frac{\partial P}{\partial \varphi }\right)}^{-1}$$

(15) P(q →0 )=1

By extrapolating to a zero scattering angle, it is possible to determine the volume of the particle *V* and thus the radius of the sphere *R*. At a small *φ* ≈ 0, the derivative of the osmotic pressure ∂*P*/∂*φ* is linked to the interaction potential of droplets:

(16) $$\frac{\partial P}{\partial \varphi }=\frac{KT}{V}(1+\beta \varphi )$$

where the virial coefficient β is determined by the pair interaction potential energy *U*(*r*):

(17) $$\beta =-\frac{1}{V}\int^{\;}\left\{exp\left\{-\frac{U\left(r\right)}{kT}\right\}-1\right\}d^{3}r$$

**Figure 5:**
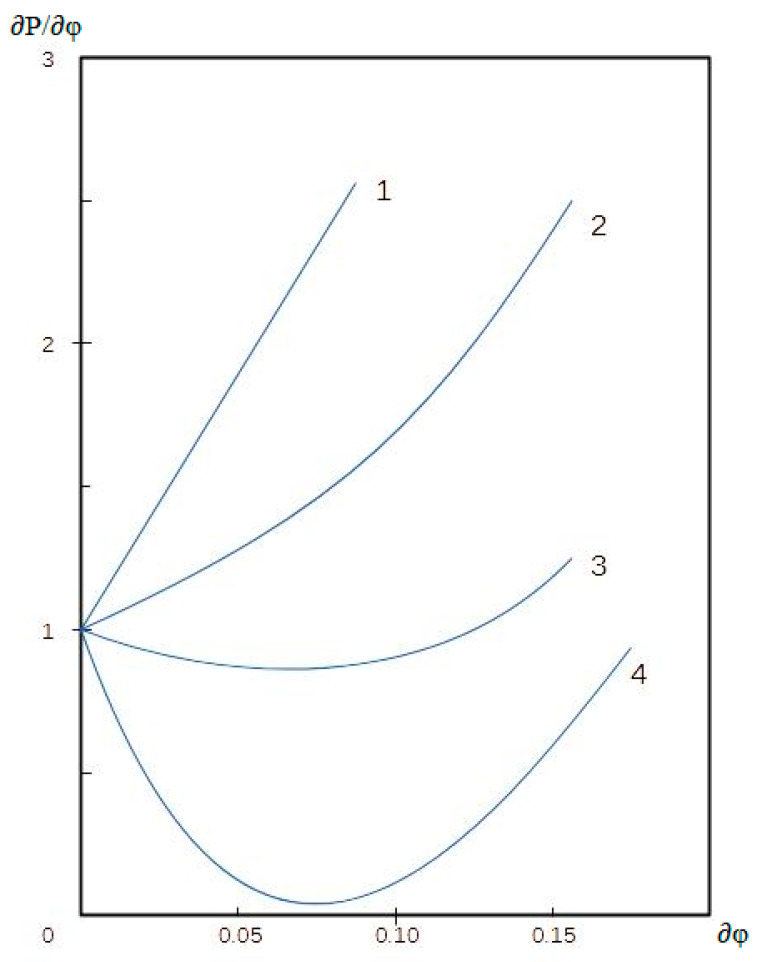
Osmotic pressure derivative curves for microemulsions. 1,2—elastic sphere, 3,4—strong potential interaction

By measuring the dependences of ∂*P*/∂*φ* on the volume fraction of particles *φ*, one can obtain important information about the interaction of drops. With spherical repulsion of drops, *β* = 8, and with attraction, *β* < 0. As can be seen from Figure 5, curve 2 characterizes the dispersion of elastic spheres, and curve 3 reflects the existence of a strong potential interaction between drops, which can be reduced by increasing the ionic strength of the solution (curve 1).

The interpretation of the light scattering data suggests the possibility of continuous dilution of the ME with the preservation of the particle size and shape, which, generally speaking, is not always true. Experience has shown [28] that in microemulsions such dilution is often realized along lines of a constant ratio of the amount of detergent in water. This means that the area of the polar part of the surfactant molecule in the interfacial layer is constant and corresponds to the formation of a saturated monomolecular film. Experiments on dynamic light scattering in the AOT–water–octane system demonstrate this regularity. In contrast to the classical scattering scheme, in the experiment on dynamic quasi-elastic light scattering, it is not the integrated light intensity I(t) that is measured, but its fluctuations in time, which are caused by the Brownian motion of particles.

The autocorrelation function of fluctuations of the scattered light *G*_2_(*t*) ≤ I(0), I(t) > 0 upon single scattering by noninteracting centers of size R < λ is related to the translational diffusion coefficient of the particle *D* by the relation:

(18) $$G_{2}\left(t\right)=1-exp\left\{-2Dq^{2}t\right\}$$

where *t* is the relaxation time and *q* is the wave factor.

In concentrated dispersions, thermodynamic and hydrodynamic interactions largely compensate each other and the determined diffusion coefficient *D* is expressed in terms of the effective hydrodynamic radius of particles *R* according to the Stokes–Einstein relation:

(19) $$D=\frac{KT}{6\pi R\eta }$$

where *η* is the viscosity.

**Figure 6:**
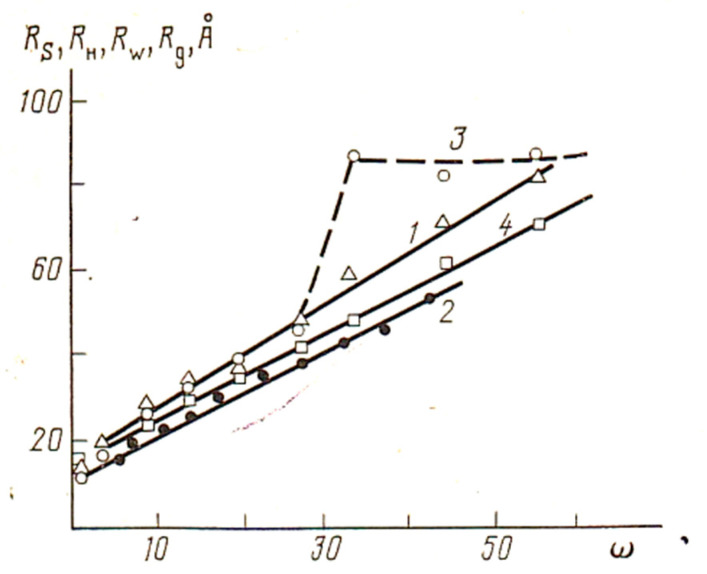
Plot of particle radius R of an AOT microemulsion in octane as a function of the degree of hydration w according to measurements by the sedimentation method and quasi-elastic light scattering [29].

By changing ω = H_2_O/AOT, i.e., the degree of hydration of the AOT molecule, one can be convinced of a linear increase in the radius of the ME particle (Figure 6).

With the extrapolation ω → 0, the droplet radius coincides with the radius of a “dry” reversed AOT micelle, which is comparable to the length of a surfactant molecule. It is noteworthy that the radius of curvature of the function G_2_(t)‚ for AOT-based microemulsion corresponds to a narrow monomodal size distribution P(_R_). Dilution of the microemulsion with the octane at ω = const does not affect the droplet radius R. To preserve the microemulsion structure, the area of the surfactant molecule (A) in projection onto the droplet surface is of decisive importance. Processing of the experimental data G_2_(t) showed that A approaches a constant value (50 Å^2^) from the moment free water appears in the drop (ω > 10). In weakly hydrated micelles, A < 50 Å^2^, since strong intermolecular interactions of AOT with water lead to densification of the surfactant surface layer and displacement of some AOT molecules into the second row. Achievement of a constant level of the A value can serve as an indicator of the distinction between the microemulsion and micellar states, which are indistinguishable by thermodynamic criteria in the phase diagrams.

The range of variation of the wave vector in light scattering 5 × 10^−4^ Å^−1^ < q < 5 × 10^−3^ Å^−1^ is insufficient to consider the details of the microemulsion structure. To study the structure of the surface layer of a drop, scattering methods with a shorter wavelength are more preferable, for example, small-angle X-ray scattering (0.01 Å^−1^ < q < 0.25 Å^−1^) and coherent neutron scattering (0.02 Å^−1^ < q < 0 < 0.18 Å^−1^). It is these methods that made it possible to prove the existence of a clearly defined interfacial surface of the ME formed by nonionic surfactants. In the absence of interparticle interference, the intensity of small-angle X-ray scattering *I*(*q*) of an isotropic sample is given by:

(20) $$I\left(q\right)=I_{e}\left(q\right)\langle {\left(\eta -\eta_{0}\right)}^{2}\rangle V_{s}\int^{\;}\gamma \left(r\right)\frac{\sin\left(q,r\right)}{\left(q,r\right)}4\pi r^{2}dr$$

where *I_e_*(*q*) is the scattering intensity for one e^−^, *V_s_* is the scattering volume and (*η* − *η*_0_)^2^ is the squared mean of e^−^ density fluctuation.

**Figure 7:**
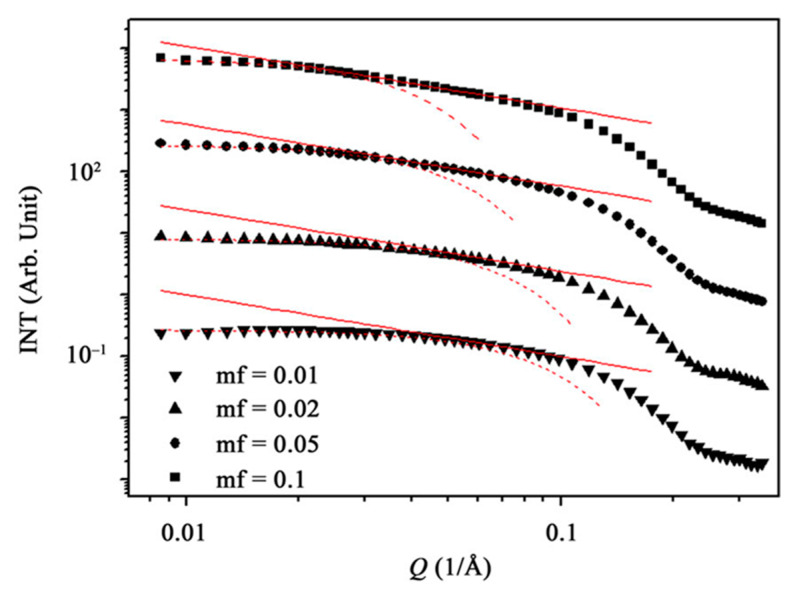
SAXS (small angle X-ray scattering) from an AOT–H_2_O–iso-octane micro-emulsion at T = 22 °C, with a constant water/surfactant ratio W 0 = 35 and vol%0 AOT + H_2_O increasing from 1.5 to 61% [30].

The correlation function of the electron density distribution *γ*(*r*) ≤ *η*(0), *η*(*r*) > η^2^ is determined by the size and statistics of the arrangement of particles in the microemulsion. The functions *γ*(*r*) for MEs based on AOT and Tween 81 were calculated by us using the inverse Fourier transformation of small-angle X-ray scattering curves (Figure 7). 

The same pattern of behavior in both microemulsions and the proximity of the position of the first minima indicates that colloidal solutions of Tween 81 in hexadecane represent a dispersion consisting of radiopaque particles of a pseudospherical shape with a radius of no more than I00 Å. Additional solubilization of water leads, as in a microemulsion, to a shift in the position of minima γ(r) towards an increase in size R. Anhydrous solutions of Tween 81 in tridecane exhibit intense scattering from particles with a correlation volume V_c_ = 4 × 10^5^ Å^3^ and a large specific surface area S/V = 350 m^2^/mL. The study showed that concentrated microemulsions of nonionic surfactant Tween 81 and anionic detergent SDS [31] give deviations from Porod’s law. The scattering intensity is described by the linear relation:

(21) $$q^{4}I\left(q\right)=Y+pq^{2}$$

where *Y* and *p* are constant.

Deviations from Porod’s law are caused by X-ray scattering on the surface of the interfacial layer formed by polar fragments of the Tween 81 sorbitan ring. Thus, the structure of microemulsions stabilized by nonionic surfactants is close to the structure of microemulsions based on ionic surfactants. This conclusion is confirmed by the method of small-angle neutron scattering, the advantage of which is the greater value of q = (0.02–0.18 Å^−1^), which makes it possible to study in detail the structure of the surface of microinhomogeneities [32]. The smaller cross section for scattering by deuterons opens up the possibility of probing individual layers of a particle by selective deuteration of surfactant molecules. Using this method, the fact of the invariability of the size of AOT microemulsion droplets in the vicinity of the critical point of delamination [32] was established, the globular structure of the particle was proved, and the shape of ME particles stabilized by ethoxylated surfactants was measured [33].

The unambiguous interpretation of these small-angle scattering methods is largely related to the problem of polydispersity. Without going into details of the special methodological apparatus, it can be stated that, for the vast majority of MEs with phase state points at the corners of the Gibbs triangle, the dispersion of particles corresponds to a narrow monomodal distribution of spherical particles. The characteristic dimensions and the Guinier radius are the averaged characteristics of a microemulsion droplet. Collisions of drops are accompanied by a small redistribution of mass and size upon impact. In the region of medium concentrations and near the boundary of the single-phase region, the polydispersity increases due to the enhancement of interparticle interactions. Thus, experimental studies of microemulsions lead to the conclusion that, at least in vast areas of phase diagrams of microemulsion systems, colloidal solutions consist of particles that have significant features of individual formations. This is the characteristic size and surface, the structure of which is clearly expressed.

## 5. Applications for Emulsions in Biotechnology and Biomedicine

### 5.1. Incapsulation and Controlled Microenvironments: Structural Investigations

Emulsions and microemulsions found their niche as a microenvironment for reactions and as a carrier for different substrates. However, this raises several questions regarding the structure of MEs and the state or processes inside the drops. The application of magnetic resonance methods details the picture of the ME structure down to the molecular level. The observation of microemulsions by the SAD method is usually carried out in the variants of measuring the chemical shift of nuclei with a change in the component composition [34], measuring the magnetic relaxation times of nuclei (T1,T2) of surfactant molecules [35], determining the self-diffusion coefficients by the spin echo method [36] and quadrupole splitting of ^2^H, ^23^Na nuclei during the formation of anisotropic liquid crystal structures. Spectroscopy is sensitive to the chemical structure of surfactants. For example, ^13^C-NMR showed that Tween 81 is a heterogeneous product, consisting mainly of two fractions, the molecules of which differ in the place of attachment of hydroxyethyl groups. Apparently, many surfactants of commercial origin are obviously heterogeneous, and the use of NMR spectroscopy is appropriate for a preliminary conclusion about the purity of the product. The latter is essential for the theoretical interpretation of the results of the study of microemulsions, since it is known that the presence of impurities can change the boundaries of the phase regions of the systems under study.

**Figure 8:**
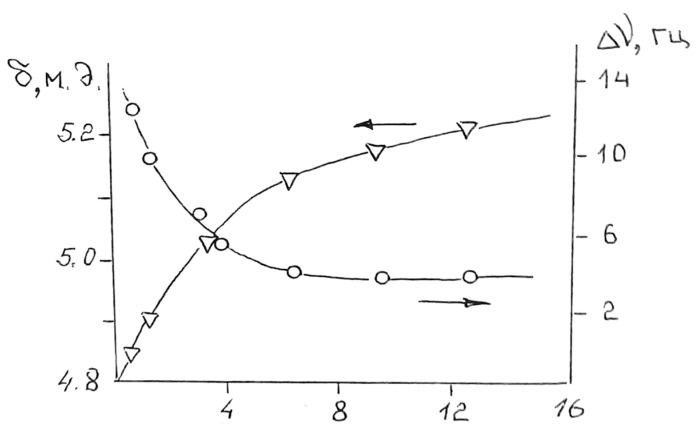
Concentration dependence of the position and width of the ^1^H-NMR proton resonance line in a Tween 81 microemulsion in hexadecane [40].

The methods of ^1^H-NMR spectroscopy in studying the hydration of surfactants and MEs can serve as a criterion for distinguishing between MEs and micellar systems. Figure 8 shows the results of measuring the position and half-width of proton resonance lines (∆ν) of water in a microemulsion formed by a mixture of Tween 81-H_2_O-C_16_H_34_ at 20 °C by the 1H-NMR Fourier spectroscopy. The curves clearly show the dynamics of hydration of hydroxyethyl groups with a monotonic shift of the NMR line to a weak field, which is interpreted as the strengthening of hydrogen bonds. Beginning with a ratio of −2 H_2_O molecules per CH_2_CH_2_O group, the position of the NMR line of microemulsified water corresponds to the chemical shift of water in the macrophase. This moment is the beginning of the formation of the central core of free water. With further solubilization of water, the chemical shift of protons does not change until the stratification of the system. At the moment of separation, the line broadens due to the increase in the magnetic heterogeneity of the system during macroemulsification. The possibility of spectral differentiation of fractions of free and bound water by ME by the NMR method can be used as the basis for the method for identifying w/o-type ME and conventional reversed micelles. The presence of free water in ME particles of the w/o type is an essential feature of ME, which should be considered as a criterion for separating the microemulsion and micellar states. The appearance of free water in an inverted micelle is accompanied by changes in the structure of the surface layer. Similar ^1^H-NMR measurements of an AOT microemulsion confirm the formation of a monomolecular AOT layer after the appearance of a free water core. The transverse area, which is equal to 55 Å^2^ per 1 AOT molecule in the interfacial layer, ensures the freedom of intramolecular movement of the AOT molecule. The ME bound water fraction is clearly seen in the ^1^H-NMR spectrum after free water has been frozen out. The sharp difference in the width of the NMR line of ice (~100 kHz) and water (~1 Hz) makes it easier to observe the NMR line of the bound nonfreezing fraction of water in high-resolution spectra. Bound water in the amount of 9 + 2 molecules per 1 AOT molecule retains molecular mobility up to −40 °C. This value of the hydrate number coincides with the sum of the hydrate numbers of all hydrated groups and the Na^+^ ion, COO^−^. Microemulsions based on ethoxylated surfactants contain bound water due to hydration of CH2CH2O groups with two water molecules. Hydration of the Tween 81 molecule, according to ^13^C-NMR data, occurs along ethoxylated units without affecting the sorbitan ring [37,38,39].

The localization of water in the drop structures of microemulsions affects the measured molecular diffusion coefficient DS of water when observing its trajectory over times t comparable to the lifetime of a drop. Comparisons of the DS coefficients of H_2_O, surfactant and oil molecules by NMR spin echo measurement provides a unique way to test the droplet model of the microemulsion structure. Lindman’s work [41] established that triple MEs with AOT detergent actually have a droplet structure. In the entire single-phase region, the self-diffusion coefficients DS of AOT molecules and are almost equal to each other because their mobility is entirely determined by the speed of the drop. As the droplet size R increases as water solubilizes, ω decreases in value.

In contrast to microemulsions based on AOT, no drop structure was observed in four-component microemulsions stabilized with alcohol codetergents. For such systems, the key element of the packing of molecules in the interfacial layer is the so-called “packing” ratio ν/al, which determines the radius of curvature of the surface layer [42]. The value of ν/al depends on the volume of the hydrocarbon part of the molecule ν, the diameter of its polar part a, and the length of the molecule I. The theory is to expect:

1. Ordinary micelles at ν/al < 1/3;
2. ME type o/w at 1/3 < ν/al < I;
3. ME type w/o at ν/al > I.

At ν/al = 1, the microemulsion acquires a transitional structure, presumably of the lamellar type, which is formed in the region of the w/o–o/w inversion. The ME interfacial layer is a highly labile structure. The data of spin probing of the Tween 81 microemulsion layer using hydrophobic radicals of the oxy derivatives of N-oxyl-oxazolidine testify to the high mobility of the hydrocarbon part of the surface and the looseness of its molecular structure. The EPR spectra of the solubilized probes in the ME phase coincide in appearance with the spectra of their solutions [40]. The correlation time scale tc~1 nsec and the smallness of the anisotropy parameter S~0.61 indicate a low rigidity of the surface layer of microheterogeneities. The value of the anisotropy parameter S makes it possible to interpret the structure of the interfacial layer as a wavy surface with a modulation period of 10–100 Å. The surface stiffness factor K is slightly higher in the so-called birefringent microemulsions with ν/al = 1. Studies of the relaxation times of ^13^C nuclei in a microemulsion based on Tween 81 [38] are in good agreement with the results of spin probing, indicating a high segmental mobility of hydrocarbon moieties surfactant molecules in the surface layers.

**Figure 9:**
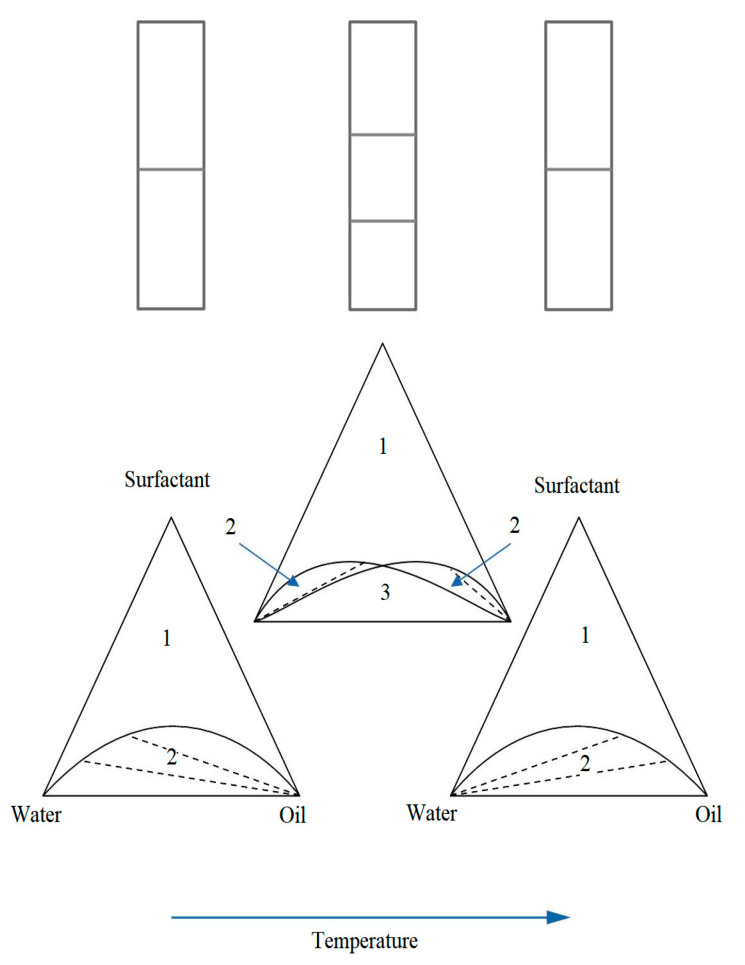
The position of the phase points on the phase diagram corresponding to the formation of three types of Windsor microemulsions. 1—isotropic phase, 2—two phase, 3—ternary phase.

According to the nomenclature proposed by Winsor [43], microemulsions are able to be in thermodynamic equilibrium with an excess of oil, water, or both components at the same time, which, according to the types of equilibrium, are called Winsor I, II and III microemulsions and their derivatives (Figure 9). Winsor microemulsions, when dispersed, form stable concentrated macroemulsions. Transitions between Winsor microemulsions are possible upon heating or the introduction of electrolytes into the system. Winsor III type microemulsions are a three-phase system in which the microemulsion phase, called the middle phase, is simultaneously in equilibrium with oil and water [44]. Such equilibrium diagrams are typical for mixtures of three pairs of partially miscible liquids. 

**Figure 10:**
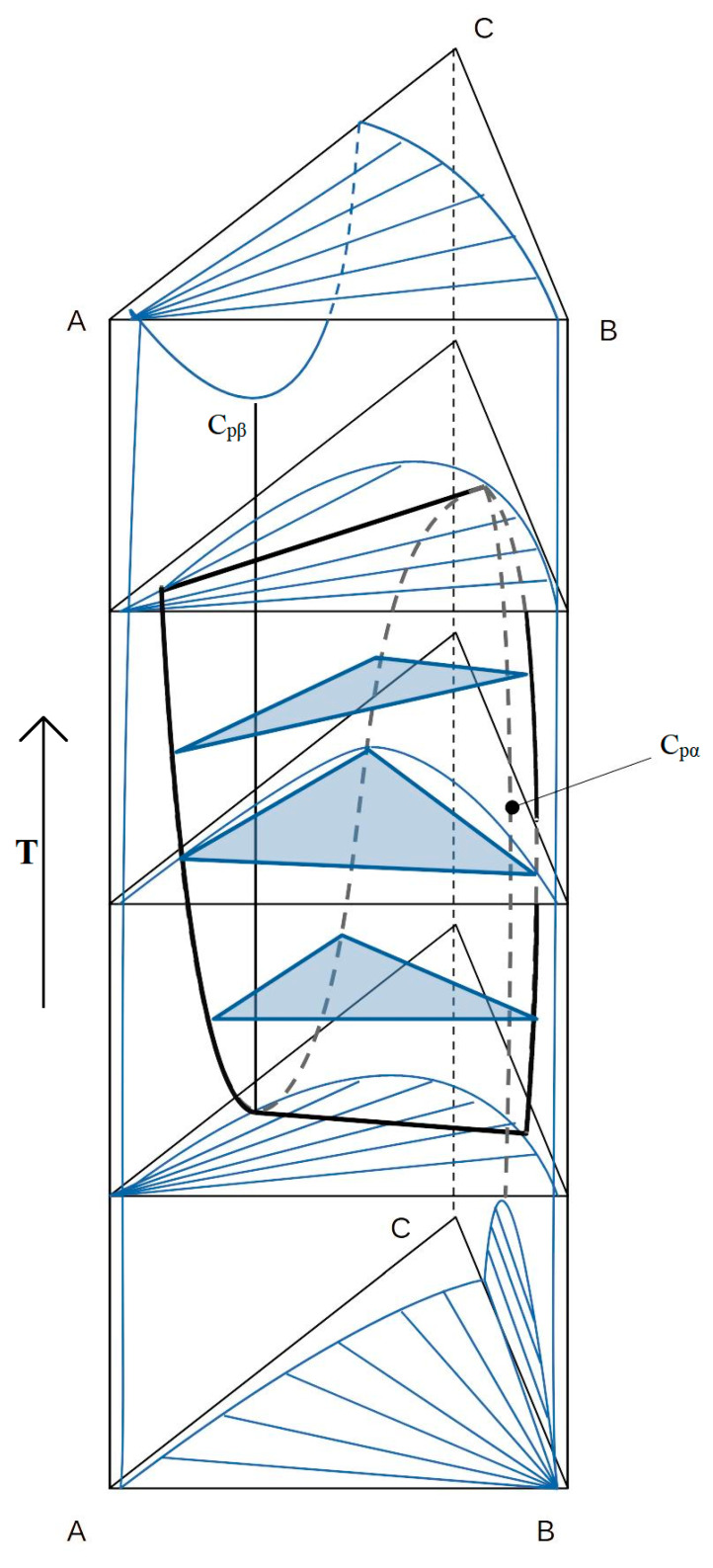
Temperature behavior of the phase diagram of the triple system oil (**A**), water (**B**) and oxyethylated hydrocarbon (**C**). Cpa and Cpß are critical stratification lines.

In practice, phase diagrams are built by determining the phase state along the lines of constant ratios of surfactant/H_2_O or surfactant/oil. In this way, the vast majority of diagrams of multicomponent systems [45] are constructed. As an example, Figure 10 shows the temperature section. prisms along the line of constant ratio CiE/H_2_O of the triple system H_2_O—CiE–oil. The diagram has a lower two-phase region of Winsor III, a region of three-phase equilibrium of the middle phase, and an upper region of Winsor I. Along the edges of the diagram, narrow ribbon zones of isotropic phase I are visible. surfactant. By a good choice of state variables, one can reach the triple critical point, at which the difference between all three phases disappears [45,46,47]. The appearance temperature of the middle phase, other things being equal, is determined by the HLB of the selected type of surfactant.

When dispersing two-phase microemulsions of the Winsor III type, stable macroemulsions of water or oil with a high degree of dispersion are formed. An important factor in the stabilization of such emulsions is the reduced surface tension γ at the interface in the presence of the microemulsion phase. As a result of a theoretical consideration of the nature of the ultra-low value γ ~ 10^−2^–10^−4^ dyn/cm, it was found that for Winsor microemulsions I and II, the surface tension is determined by the structure of the monomolecular surfactant film separating excess oil or water from the microemulsion. For dilute Winsor microemulsions I and II, a low value follows logically from calculations of the minimum free energy of the system at thermodynamic equilibrium of a microemulsion with a dilute micellar phase [42]. According to the DLVO theory, the opposite direction of the influence of potential interactions between particles and the entropy mixing factor determines the low interfacial energy of the system. In the Talmon-Prager models, the low value of γ as the leading reason for the thermodynamic stability of emulsions I and II is given a special meaning. Measurement of such low values in the range of 10^−3^–10^−4^ dynes/cm presents notable difficulties. The most acceptable methods are the rotating drop method and light scattering from the liquid surface. When considering the behavior of interfacial tension γ on the cross sections of phase diagrams of ternary systems depending on temperature or ionic strength, a strong decrease in γ is found in the region of an increased content of detergent in the microemulsion, i.e., in the middle phase. A typical example of the behavior of γ when passing through the middle phase of a microemulsion is shown in Figure 10, which displays the temperature behavior of the interfacial tension coefficient γ at the oil–ME (γmo), water–ME (γwm), and water–oil (γow) interfaces. The data of light scattering and measurements of self-diffusion coefficients confirm the closeness to the critical state of the system in the region of the observed decrease in γ in the Winsor III microemulsion. The strongest drop in the value of γ is observed in the vicinity of the critical points of delamination.

In a three-phase region, the Antonov rule is not observed, but the following inequality is satisfied:

(22) $$\gamma_{ow}<\gamma_{mo}+\gamma_{wm}$$

It can be expected that as we approach the triple critical point delamination, the surface tension will be negligible. Indeed, in the vicinity of these points, the system is described by a number of critical exponents; in particular, the coefficient γ obeys the law γ = γ_o_ ε^2^ν, where ε is the relative deviation of the temperature T from the critical Tc and ν is the critical exponent of the divergence of the correlation length ξ. In microemulsions of anionic detergents with a “drop” structure, in most of the single-phase region, approaching the critical separation surface is accompanied by an increase in fluctuations in the concentration of globular particles without changing their size. Microemulsions based on AOT are described by the model of van der Waals interaction of spheres with a small potential of attraction between the spheres, which provide the skewing law of critical behavior ξ with index ν = 0.75 [45]. The critical state of the middle phase of microemulsions was also revealed in microemulsions of nonionic surfactants, alkyl sulfates, ammonium salts, etc. The passage through the middle phase can be carried out not only by heating, but also by varying the electrolyte concentration in the microemulsion. The critical state observed in micro-emulsions has a number of important features that distinguish them from critical phenomena in ordinary liquids. The first feature is the expansion of the temperature interval for the development of critical fluctuations to several degrees, the second is the variety of ways to achieve the critical state of systems by selecting the composition, type of emulsifiers, ionic strength, etc. As heating increases, the surfactant solubility in oil increases and the volume fraction V of emulsified water increases in accordance with the Bancroft rule. At temperatures below the inversion point, a reversal of the pelvis is observed with the appearance of stable emulsions of the o/w type.

As a result of multilateral studies of the conditions for stabilizing emulsions using microemulsions based on non-ionic surfactants, it was possible to formulate several methodological rules:

1. The phase inversion temperature in emulsions is proportional to the HLB of the emulsifier used;
2. The greatest dispersion of the emulsion is achieved in the vicinity of the emulsion inversion temperature;
3. The HLB of ethoxylated adducts used to stabilize emulsions increases with an increase in the number of CH_2_CH_2_O groups in a surfactant molecule;
4. The phase reversal in the emulsion with the participation of the microemulsion phase passes through the formation of an average micellar phase and is accompanied by a sharp decrease in the interfacial tension in the system.

The conclusions drawn remain valid to a certain extent when replacing water in microemulsions with some other polar solvents such as glycerol, alcohols or molten salts [48,49,50].

Among the unique properties of microemulsions, which are of great importance for the implementation of biochemical reactions, is a high solubilizing ability with respect to molecules of different hydrophobicity and size. At present, great success has been achieved in the solubilization of ionic and non-ionic nature of native proteins by microemulsion systems, which was the basis for the construction of micellar enzymology, the priority in the creation of which belongs to the Soviet school of researchers led by I.V. Berezin. Colloidal enzyme systems combine the biocatalytic advantages of aqueous solutions of enzymes with the possibility of selective extraction of hydrophobic substrates from hydrophobic solvents. The principal result obtained in the study of microemulsions on the basis of AOT is the ability of these microemulsions to include proteins inside microemulsion particles without disturbing the protein structure. The optimum catalytic activity of most enzymes in droplet microemulsions corresponds to the condition of geometric similarity between the protein and the particle. In some cases, the phenomenon of an increase in the activity of enzymes in microemulsions (for example, 2-chymotrypsin in AOT microemulsion) was noted in comparison with aqueous solutions. The denaturing effect of surfactants on proteins is minimal in microemulsions containing a water core of a suitable size. The syllable of hydrated water of the polar parts surfactant molecules shield the protein from contact with the denaturing agent. When the size of the protein is larger than the characteristic size of the ME droplet, the protein induces around itself a micellar core with a shell of hydration water. During the solubilization of membrane proteins of increased hydrophobicity, protein molecules can be adsorbed on the surface of the droplet. Depending on the type of protein and surfactant, various variants of localization of macromolecules inside reversed micelles can be realized. Apparently, one can expect the inclusion in reversed micelles of not only proteins, but also structures of a higher structural hierarchy, such as DNA, viruses or individual cell organelles, and hope for the possibility of inclusion in microemulsion systems. It should be noted that the known experimental material on micellar enzymatic catalysis refers to microemulsions with a composition close to the peaks of the phase diagrams of multicomponent water–oil–surfactant systems. Questions of protein solubilization in the middle phase, temperature stabilization, and aggregation stability of microemulsified proteins in critical regions and regions of phase transformations of one of the components remain open for future research. One of the central problems of biotechnology is to find ways to prolong the action of biologically active substrates or intact cells. The solution to this problem is usually achieved by drying the drug or storing it at low temperatures. The main obstacle to successful cryopreservation is the inactivating growth factor of large ice crystals and the hyperconcentration of salts during freezing. The main ways to combat the adverse effects of low-temperature ice crystallization are reduced to the search for a cryoprotective type of polyethylene glycol solutions, TMC0, which lower the freezing point of water. The ability to supercool water in the microemulsion state opens up a new independent way to solve this urgent problem. As was shown in our experiments on low-temperature measurements of NMR spectra [38,40], the bound water of microemulsions based on Tween 81 and AOT emulsifiers easily tolerates cooling below −50 °C without ice formation, if the water content does not exceed the surfactant primary hydration threshold. The free water of microemulsions, being localized in a small volume, is easily supercooled below −10 °C. Protein molecules embedded in the cavities of microemulsion droplets are not nucleation centers, which allowed development of biocatalytic systems based on colloidal solutions of enzymes that function at subzero temperatures without denaturation [51]. In such systems, use silicone oil with a low viscosity and a freezing temperature of −50 °C as a hydrophobic solvent. New cryopreservation media are available for the penetration of oxygen, and substrates of various natures were used to simulate the operation of redox chains involving cytochrome C, peroxide and bacterial luciferase.

Reversed micelle systems contribute to the stabilization of enzyme preparations, providing extended shelf life over a wide temperature range. The use of organic solvents with a melting point of 20 °C is not an obstacle to maintaining the activity of the drug, because the hydrated environment of the protein included in the micelles does not allow the contact of the protein with the hydrophobic surface of the organic phase, regardless of its state of aggregation. Our observations showed that when the structure of microemulsions is destroyed after the crystallization of the hydrophobic solvent C_16_H_30_ (T_melt_ = 18 °C), the emulsifier molecules (Tween 81) are displaced by the crystallization front without noticeable perturbation of the structure of hydrated water. Apparently, the adsorbed layer of the emulsifier has many similarities with the surface layer of the detergent in microemulsion drops. After paraffin melting, spontaneous microemulsification is observed, which is the premise for recommending such systems as possible preparation storage media. A large degree of dispersion of the material after melting is an advantage of such systems in comparison with conventional non-emulsion type cryostabilizers. The list of substances solubilized by microemulsions is extremely diverse. In the microemulsion state, solutions of monomers were obtained for carrying out reactions of radical polymerization and polycondensation. The selective incorporation of monomers and biologically active substances into the cores of Winsor I-type droplet glycoemulsions served as the starting point for the creation of microencapsulation methods, which make it possible to synthesize products of the nanometer range of a spherical shape (0.01 μm).

The aggregative stability of microemulsions containing polymer cores is provided by a monomolecular layer of surfactant (in example, cetyltrimethylammonium bromide with hexanol as a codetergent) covering the polystyrene core. Encapsulated polymer emulsions can be diluted with a solvent while maintaining the original particle size, which distinguishes microemulsions from conventional three-component microemulsions, which can be diluted only along certain lines of the phase triangle section. In particular, the isoline of constant size in the AOT microemulsion runs along straight lines with a constant H_2_O/AOT ratio. The ability of microemulsions to microsegregate reaction components is widely used in the creation of microreactors for reactions that require a spatial separation of the initial reagents and products. In connection with the urgency of the task of switching energy to environmentally friendly energy sources, the reaction of water decomposition with the help of light received attention. The incorporation of catalysts (such as ruthenium complexones) into microemulsions makes it possible to carry out reactions in a controlled microenvironment.

### 5.2. Microemulsions in Burn and Wound Treatment

Unique physical and chemical properties of nanoscale multiphase liquid compositions, such as colloidal stability, low surface tension, biocompatibility and solubilization of poorly soluble substances in water, are studied in detail from the angle of practical application in the technology of microencapsulated drugs, creating methods for targeted delivery of pharmaceuticals to affected organs, improving the diagnostics of oncological diseases, and the creation of innovative transdermal agents for the treatment of local and systemic lesions. A brief list of practical applications of nano- and microemulsions in various fields of biomedicine is presented in Table 3. The increased demand of medicine for the development of new, effective vaccines that prevent the spread of infectious diseases of measles, tuberculosis, HIV and COVID-19 has directed research towards the use of micro- and nanoemulsions as promising adjuvants in various options for the application of nasal, oral and inhalation forms of drug administration. The ability to enhance solubilizate incorporation into cells in emulsion nanoparticles is being studied in detail as an alternative treatment for diabetes with emulsified insulin or intermediates in the glucose uptake receptor mechanism in type 2 diabetes. Micro- and nanoemulsions are the basic platform for the manufacture of a wide range of dietary supplements due to their compatibility and ability to absorb large amounts of essential fatty acids, vitamins and important amino acids [52,53]. The advantage of such nutritional supplements based on nanoemulsions is their high absorption capacity with respect to lipophilic compounds such as carotene (provitamin D). Carotene nanoemulsions are successfully used in veterinary medicine to prevent the syndrome of night blindness in animals. Antibiotics in the form of nanoemulsion preparations exploit the important property of increased retention of poorly soluble compounds to increase the dose and prolong the action of the antibiotic in the gastrointestinal tract. The use of the antibiotic Rifampicin in a nanoemulsified state expands the possibility of treating tuberculosis with aerosol applications and transdermal ointments in the long term. Vancomycin and gentamicin have a wide spectrum of antimicrobial activity as part of microemulsions based on ethoxylated fatty acids. The nanoemulsion forms of antibiotics allows monitoring of the release of the bactericide agent into the bloodstream of the lymphatic system, improving the pharmacokinetic profile of the drug. A wide range of changes in the viscosity of multiphase emulsion systems in dependence on content of surfactant and cosurfactant opens up ways for flexible drug management when applied externally in wounds or trophic ulcers in the form of ointments and aerosols. The use of oils based on bioactive lipids as a hydrophobic phase provides additional therapeutic benefits.

The management of local anesthesia in surgical interventions, treatment of teeth, burns and chemotherapy (skin irritation) is an important task and can be carried out by using anesthetics in microemulsions. The inclusion of lidocaine together with K3PO4 in a microemulsion stabilized with Pluronic127 surfactant in Capmul MCM C8 EP oil provides an increase in the duration of anesthesia with percutaneous administration [65,66].

Topical application of anti-inflammatory drugs is an important and extensive area of pharmacology. It is known that nanoscale dispersion of ibuprofen, aspirin and a number of other non-steroidal anti-inflammatory drugs increases the effectiveness of their action depending on the type of emulsion and etiology of disease pathology. An increase in viscosity and a transition to a gel state improves the application of the drug in wounds by holding the pharmaceutical substance at the treatment site. A variety of formulations of multiple phase systems based on ethoxylated and lipid emulsifiers have been proposed in various stages of clinical application [67,68].

The ability of microemulsion compositions to combine with polysaccharides without significant perturbation of the phase equilibrium has been most thoroughly studied for the case of widely known natural polysaccharides such as chitosan and hyaluronic acid. Chitosan is widely used in various fields of technology and medicine as a carrier of pharmacological substances, the basis of medical products [69]. The applied use of chitosan in biotechnology and medicine is facilitated by its low toxicity, biocompatibility, biodegradation and availability of raw materials. The bulk of dispersed chitosan in pharmacology is used in the form of micronized forms—microspheres with a size of about 1-100 microns [70]. In connection with the increased interest in the development of targeted drug carriers and diagnostic tools, a problem arose in creating stable nanodispersed forms of chitosan that can include proteins and diagnostic reference molecules or colloidal adducts and subsequently ensure their intracellular targeted delivery. To maintain nanodispersions in a stable state, it is necessary to reduce the free energy of mixing, primarily, the interfacial surface energy. The latter, as a rule, is achieved by involving surfactants of an amphiphilic nature in the nanoemulsification process. With a successful composition, it is possible to obtain thermodynamically stable nanoemulsions with a particle size of up to 1–10 nm. In this way, nanodispersed inverse w/o emulsions stabilized with the non-ionic detergent Tween 81 were obtained, which are able to solubilize enzymes, polysaccharide macromolecules, and even include individual cells and organelles in the composition of multiple emulsions [71,72,73].

The current state of work on chitosan is characterized by the focus of efforts towards the creation of suture materials, dressings impregnated with medicinal enzymes. Most of the information on the application of chitosan refers to coarse chitosan or its solutions and is reflected in the abundant patent and monographic literature [74]. Among the main directions, one can single out the development of delivery vehicles for anticancer drugs such as doxorubicin, cis-platinum, etc. [75]; bioadhesive microencapsulated chitosan for delivery of hormonal proteins such as insulin, tumor necrosis factor, calcitonin, etc. [76,77]; and biodegradable suture materials such as wound dressings and sponges. In the development of new materials, a direction in the synthesis of modified and composite materials stands out. PEGylated and hydrophobized forms of chitosan have been obtained, which aggregate to form microspherical nanoparticles and nanocolloids [78,79,80] There are new studies in the development of magnetic composite materials based on chitosan for the suppression of cancerous tumors by hyperthermia [81,82,83]. The fundamental possibility of synthesis and compatibility of superparamagnetic nanoparticles of iron oxides was shown in [84,85,86]. A distinctive feature of most studies on nanodispersed forms of chitosan is the empirical search for the formula of the material and the absence of a targeted study of the molecular structure and intermolecular interactions in the nanocomposite. Hyaluronic acid (HA) has found wide application in cosmetology and dermatology as a corrector of epidermal pathologies, for maintaining water balance, and for treating arthritis of the joints. Biocompatibility, non-immunogenicity, viscoelasticity and compatibility with pharmacological agents have identified HA as an integral component of skin improvement formulations, implant material for osteoarthritis and as the basis of ophthalmic drugs [87,88,89,90,91,92]. 

In the cause of nanoemulsification, HA forms emulgels, in which nanoparticles, while maintaining a small size, form a macromolecular network with a high water retention and active pharmaceutical substances. Hyaluronic gels have found wide application in dermatological practice [93,94,95,96]. The introduction of geroprotective peptides—Thymogen (Glu-Trp), Vilon (Lys-Glu) and Epitalon (Ala-Glu-Asp-Glu)—into hyaluronic emulgels enhances the pharmacological properties of such compositions. The peptide component stimulates the metabolism of the connective tissue and enhances the reparative response of the body, accelerates the healing of the affected skin and mucous membranes. As part of the emulgel, the bioactive peptide is in the form of a complex stabilized with hyaluronic acid. The preparation implements the conditions for the solubilization of the peptide in intact form and the prolongation of its action due to the original micellar–gel structure. The next step in the development of the concept of enhancing the bioactivity of nanoemulsions was research on the inclusion of immunoactive proteins and nucleotides in compositions that turned out to be adjuvant vaccine formulations [97,98].

The actual direction of application of nanosized forms of chitosan, hyaluronic acid in emulsions is non-invasive diagnostics of pathogenetic changes in the human body. The inclusion of quantum fluorescent and magnetic dots in nanoparticles formed during emulsion dispersion expands the scope of diagnostic applications of MRI and confocal microscopy for early cancer diagnosis. The principle of operation of such diagnostic systems is determined, on the one hand, by the special physical properties of metal or semiconductor nanoparticles that are part of the nanosensor element, and, on the other hand, by the ability of marker nanoparticles to remain unchanged when they enter the zones of the visualized area of pathogenesis. The central task of designing non-invasive diagnostic systems is to obtain stable nanosized particles with a monomodal distribution and a given chemical structure. The existing methods for the synthesis of nanoparticles are diverse, but have their limitations. For example, the production of nanosized elements, quantum dots by the method of epitaxial growth on the surface gives particles of the correct shape with a small size spread. The quantitative yield of nanodispersed products gives synthesis in multiphase water–organic mixtures, but the technology of synthesis in nanodispersed media is sensitive to the structure of nanoparticles and the requirements of a specific clinical application of the product. Depending on the recipe composition of the reaction media and synthesis conditions, nanoparticles can be obtained in the form of irregularly shaped formations with a wide size distribution. Preservation of the biological activity of ligands is an additional requirement for choosing the optimal synthesis technology. As a result of testing various technological approaches, it was found that the methods of ionotropic gelation and coacervation in layers in solutions of chitosan polysaccharides provide a kind of phase behavior of multicomponent systems with the release of a nanodispersed product [99,100,101]. The size distribution of nanochitosan particles during ionotropic gelation is very sensitive to the ratio of components in the mixture, temperature, and changes over time. The reasons for the complex phase behavior lie in the variability of intermolecular interactions during nucleation and phase decomposition and were poorly studied before the use of NMR spectral analysis methods that were not subject to the limitations of the optical turbidity of the medium.

Aside from chitosan nanoformulations, albumin nanoparticles have found their use in biomedicine. Modification of albumin sites with hydrophobic ligands leads to a nanoparticle self-assembly. This strategy allows for drug encapsulation combined with other diagnostic or therapeutic drug delivery, useful in non-invasive treatment (for example, photodynamic therapy) [102]. 

The MRI method, which operates on the principles of nuclear magnetic resonance, is widely used in clinical medicine to record pathological changes in organs. The information content of MRI diagnostics increases sharply when using contrast agents of paramagnetic elements of gadolinium and superparamagnetic nanoparticles of iron oxides. The inclusion of a magnetic reference and a targeted bioligand into the composition of the nanocarrier increases the diagnostic ability of the system. Due to its biocompatibility and the presence of active amino groups, nanochitosan is a convenient matrix for incorporating physical labels into a carrier for further use in diagnostics and therapy. The prospect of such use of nanochitosan for MRI is reflected in the formulation of recent work on the search for innovative contrast agents based on chitosan [81,82,84,103].

The use of an immunoactive drug in a nanodispersed form increases the bioavailability of the drug when the drug is absorbed by the capillary circulatory network and the reticuloendothelial system after the drug comes into contact with the surface of the respiratory tract or skin. The possibility of obtaining hybrid micronized forms makes it possible to combine an active immunotherapeutic agent of a protein nature and a diagnostic nanoparticle label in a single dosage form, which is observed by tomographic imaging and endogenous fluorescent optical detection during treatment. The inclusion of highly active immunoregulatory protein-cytokines in the composition of external dosage forms provides stimulation of the body’s defenses (innate and adaptive immune responses) and thereby significantly reduces the time of treatment of trophic ulcers and burns. Cytokines such as interleukin-1beta stimulate the growth and metabolism of connective tissue, ensure the supply of leukocytes to the site of inflammation and enhance the phagocytic reaction of the body, accelerating the reparative processes of complex wounds, burns and trophic ulcers [104]. The therapeutic value of such immunogenic drugs can be significantly increased by their complex use with bactericidal nanodispersed silver as part of a single dosage form, provided that the biological function of cytokines is preserved in the presence of a highly active bactericidal material. As known, the bactericidal properties of silver are widely used in medical materials. The emergence of antibiotic-resistant strains of microorganisms contributed to increased interest in the antiseptic properties of silver and its compounds. However, an increase in the content of bactericidal silver in pharmacological agents may be accompanied by side effects of damage to proteins and cellular structures of the body itself. Dispersion of silver to particles with a size in the nanodispersed range allows to reduce negative side effects while maintaining antimicrobial properties against pathogenic microorganisms. The antibacterial activity of cluster silver is almost two orders of magnitude higher than that of classical colloidal forms (protargol, collargol and poviargol) in terms of the same volume concentration of the metal. The results obtained by the authors on the preservation of the integrity of the key enzymatic proteins of cell membranes during their solubilization in microemulsion media based on non-ionic surfactants [72] proved the fundamental possibility of implementing sparing conditions for the joint inclusion of bactericidal forms of nanodispersed silver and immunoactive protein cytokines into the composition of such compositions. Microemulsion media can serve as a biocompatible basis for the creation of a completely new class of bactericidal immunotherapeutic agents, the need for the development of which is strongly dictated by the requirements of the market for pharmacological preparations for the treatment of burns and traumatic lesions of the body. The use of microemulsion media is aimed at solving important problems of creating effective antibacterial and antivirulent immunocorrective agents for the treatment of purulent wounds of burn origin, difficult-to-heal trophic ulcers, which are particularly difficult to treat due to the presence of antibiotic-resistant microflora, as well as diseases of the outer integument of the body caused by viral infections (e.g., HIV, forms of lichen, chicken pox, herpes zoster, etc.).

Local application of antimicrobial chemicals is indicated in the treatment of burns, infected wounds, poorly healing trophic ulcers and boils. The best way to use ointment dosage forms is determined by the source of infection, the type of microorganism, the degree of infection, the pathogenesis of the wound, the duration of treatment and the immunological state of the patient. As chemical antimicrobial substances, it is advisable to use agents that are simultaneously directed against bacteria, viruses and fungal disease spreaders. However, the lack of an ideal chemical preparation, the impossibility of combining antagonistic medicinal principles in one dosage form, the emergence of antibiotic-resistant strains of bacteria and allergic manifestations to many macrolides force us to reconsider the attitude towards the somewhat forgotten old Galenic drugs that have a wide range of antimicrobial activity, such as silver, mercury, zinc preparations, bismuth, etc. Traditional bactericidal forms of external use based on hydrogen peroxide, iodine, chlorhexidine, polymeric composites of iodine and a number of antibiotics are well known, as well as their limitations [105,106,107,108,109,110]. The use of traditional medicines such as honey and phytotherapeutic extracts is indicated for reasons of safety, low cost and availability of treatment for allergic skin lesions and mild thermal burns [111,112].

The currently used local treatment agents have a narrowly targeted effect, do not provide a comprehensive effect on the wound process, and are therefore ineffective as applied in a monotherapeutic regimen. The anti-inflammatory effect of antiseptic ointments and liniments can be enhanced by introducing a number of cytokines and interferons into their composition, if protective measures are taken against the inactivating action of complexing ions [113]. The latter is especially relevant in the local treatment of chronic long-term non-healing ulcers, which are the result of complications with a reduced immune status of the body against the background of viral diseases (HIV, influenza, herpes, etc.). The combined use of interferon, cytokines and antimicrobial silver as an external anti-inflammatory and antiseptic agent will expand the possibilities of therapy. Modern achievements in the field of increasing the effectiveness of silver ointment preparations are associated with the creation of hydrophobic–hydrophilic emulsion preparations of a coordinated silver ion [114,115,116,117,118,119,120]. Ointment compositions based on insoluble salts of sulfodiazine and silver thiosulfate have shown good results in the treatment of infected burns [121,122] activity of the hydrated Ag+ ion, but can be increased by dispersing silver to nanometer-sized particles. Finely dispersed metallic silver has bacteriostatic and bactericidal properties and is not subject to hydrolytic decomposition processes like silver coordination complexes. Metallic silver can be dispersed in the form of nanometer-sized nuclei in the composition of reverse micelles microemulsion compositions [123].

Microemulsified solutions of dispersed silver are capable of solubilizing low molecular weight immunoregulatory proteins in their native form [124,125,126]. The possibility of creating a hydrophobic–hydrophilic micellar composition capable of being in the gel state was shown [127,128,129,130,131]. Micellar gels included agents such as interleukin 1, recombinant interferon alpha-2b and lactamase [132,133]. The ability to include highly active silver nanoclusters, immunostimulatory proteins and peptides into microemulsion compositions is not the only condition for obtaining promising topical drugs. The practical use of microemulsion media as medicinal materials imposes a number of stringent pharmacopoeia requirements for toxicity, stability, high resorptive ability to release solubilizate, increased macroviscosity of the system to retain the material on the damaged area of the body surface, and ease of packaging on standard industrial equipment. Increasing the macroviscosity of microemulsions up to the formation of a gel by adding thickeners or increasing the molecular weight of an organic solvent is not always possible, since it can lead to a violation of the phase state of the composition as a whole.

**Figure 11:**
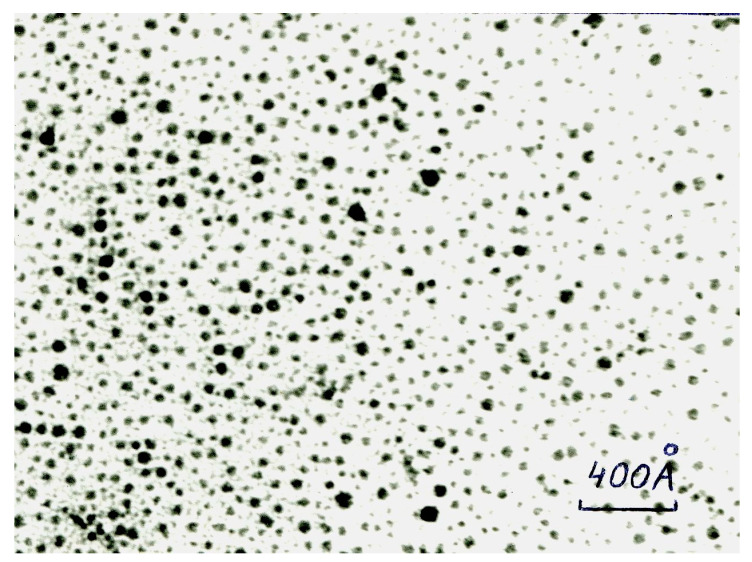
TEM image of silver nanoparticles in the Intergel composition [134].

An example of an ointment composition of the combined immunoactive and bactericidal action of a nanoemulsion structure is a nanoemulsion with the code name “Intergel” [134]. Intergel is composed from water nanoemulsion stabilized by oxyethylated adduct of stearic acid and oxyethylated phenol in vaseline oil. Topical antibacterial nanoemulsion ointment “Intergel” loaded by nanoclusters of silver and immunoactive protein interleukin-1β (IL-1β) was developed by nanotechnology means. Ointment consolidated from silver nanoparticles entrapped into surfactant gel. TEM image of ointment demonstrate nanoformulated structure Figure 11.

**Figure 12:**
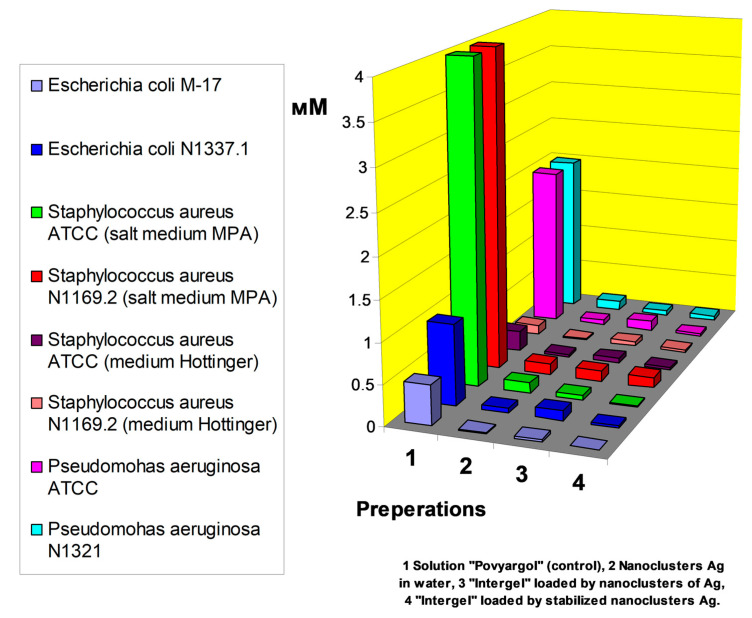
Diagram of relative bactericidal action of silver in different compositions estimated by minimal bactericide concentrations of Ag [134].

A peculiar property of nanoemulsion “Intergel” is its ability to form a stable gel at normal temperature. The gel formulation was designed for treating burns and trophic ulcers in hard cases of immunosuppressive etiology. The formulation has a great potential to enhance absorption of immune mediator IL-1β by damaged tissue. Burn and trauma infection are proportional to the extent of injury and generalized immune suppression. Antibacterial activity against Gram-positive and Gram-negative strains *Escherichia coli* M-17, №1337.1, Staphylococcus aureus ATCC-№25923, №1169.2, Pseudomonas aeruginosa ATCC-№27853, №1321, Enterobacteriaceae, Micrococcaceae and yeast-like fungi from clinical isolates and museum collection were assayed compared to commercially available pharmaceutical “Povyargol” (Ag/poly(N-vinyl—2-pyrrolidone composite). The results reveal high bactericidal and fungicidal activity of formulation to wound microflora are shown in Figure 12. Cytotoxicity of ointment to somatic cells was assessed on cell proliferation of thymocytes. There were no indications of toxic influence to somatic cells.

**Figure 13:**
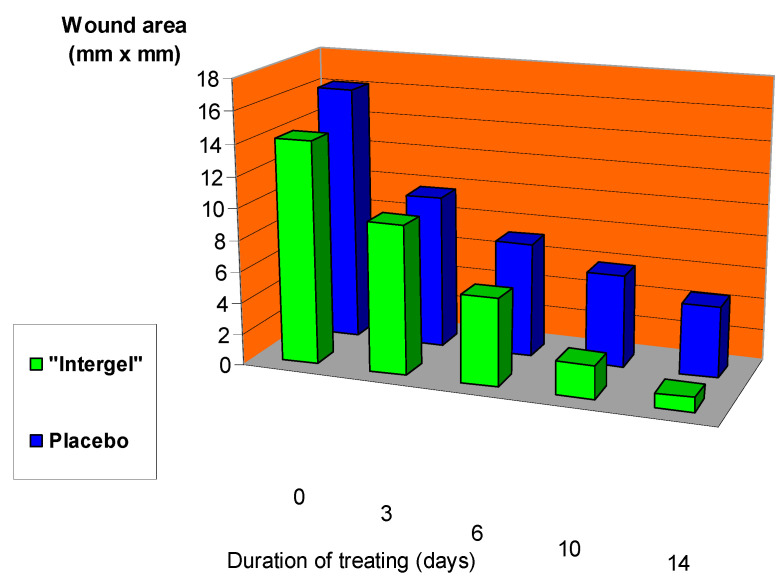
Contraction of wound area of trauma in mouse model under treatment by nanosilver formulation supplemented cytokine IL-1 beta [134].

The wound healing activity of Il-1β applied in the form “Intergel” was confirmed in a immune-compromised mouse model when the immunity had been suppressed by hydrocortisone administration. The ointment “Intergel” was applied to mice with skin surgical injuries once a day. Application of “Intergel” ointment caused the reduction of wound area, acceleration of formation of new epithelium, and increased the rate of granulation process. Furthermore, the topical treatment by “Intergel” ointment was efficient in healing of skin trauma in animals. Contraction of wound area following application of “Intergel” ointment in the experimental mouse model is shown in Figure 13. The nanocrystalline silver is considered effective against broad-spectrum wound infections. The presence of nanoclusters silver particles facilitates immunostimulatory and wound-healing activity of preparation in topical application of IL-1β. Therefore, a nanoemulsion system is convenient to sustain immune-mediator Il-1β and bactericidal silver in one gel-like composition without loss of activity preparation by nanotechnology method. A nanosilver composition comprised Il-1ß was found to be beneficial for improving the healing of trauma.

The current gel nanoemulsion compositions have the advantages of nanocarrier emulsions and gels for treatment of burn wounds processes [135]. Nanoemulsion gel formulation must be optimized for burn wounds through analysis of rheological and sensory properties. The gel state gives rise of sustained drug immobilization in damaged skin areas. The latter play a great role in post burn care [136]. The usual treatment of a burn wound includes three stages [137]. The first stage is the treatment without a dressing, the second is treatment with a dressing saturated by pharmacological ointment, and the last stage is the reconstruction of skin and the repair of scars [138,139]. The most popular formulae for microemulsion treatment in medicine and cosmetological practice are emulsified lotions, balsams, gels, ointments, aerosols and modern emulgels [140,141]. According to the last reports, the new innovative nanoemulgels are developed as a prolonged release drug system [142]. These recipes have a necessary time for the therapeutic duration and minimal restrictions related to adverse reactions of medicinal substances and base components of formulation. Skin care after burn damage demands long term, consistent application of pharmaceutical medication in various formulations. In current medical treatment of first degree burn wounds, the following substances are offered for effective usage [143]: hexyl laurate, PPG-15, stearyl ether, Butyrospermum Parkii (shea butter), glycerin, steareth-2, steareth-21, dimethicone, panthenol, menthyl lactate, tocopheryl acetate, calcium gluconate, acrylate/sodium, aqua, olea europaea fruit oil, sorbitan olivate, cetearyl olivate, panthenol 75%, isostearyl neopentanoate, myreth-3 myristate, Scutellaria Baicalensis root extract, sorbeth-30, squalane, Silybum Marianum extract, carbomer, sodium hydroxymethylglycinate (50%), menthol, sodium hyaluronatacryloyldimethyl taurate copolymer, isohexadecane, polysorbate 80, cetearyl alcohol, phenoxyethanol, methylparaben, ethylparaben, propylene glycol, diazolidinyl urea, benzyl salicylate, butylphenyl methylpropional, citronellol, limonene, linalool, benzyl benzoate, geraniol, benzyl alcohol and sodium hydroxide [94]. Acceleration of wound healing by stimulation of regeneration of the epidermis is achieved using creams based on Glycine Soja (soybean) oil, Simmondsia Chinensis (jojoba) seed oil, Cera Alba, Vitis Vinifera (grape) seed oil, Hippophae Rhamnoides (seabuckthorn) berry oil, betulin, sodium stearate and citric acid. The products containing such substances give satisfactory result of skin care [144]. The obtained nanoemulgels of these substances have shown apparent viscosity, consistency, reabsorption of drugs and adhesion to the wound basement. Good hydration of burned skin is associated with the gel state of these preparations with a high content of moisturizing ingredients, such as propylene glycol, glycerin, symphytum officinale extract, panthenol, allantoin, hyaluronic acid and aloe vera gel (in the case of the nanoemulgel) [145]. Nanoemulgel on the base of sea buckthorn (Hippophae rhamnoides) berry oil stimulates regenerative processes in the epidermis and supports wound healing due to palmitoleic acid (ω − 7,) a similar component to skin lipids [146,147,148]. The most important moisturizing substances presented in microemulsion systems are dexpanthenol, allantoin, aloe vera gel [149] and hyaluronic acid [54]. Allantoin stimulates epithelization accelerating the regeneration process of damaged and inflamed skin. Thanks to allantoin, the proliferation of epithelial cells is accelerated, which enhances the recovery of skin damage [150]. Allantoin induces the skin to retain additional water, and can restore the hydro-lipid surface. Pharmaceutical and cosmetic products with allantoin are also recommended for the treatment of chronic diseases such as ulcers, hard thermal burns, atopic dermatitis and psoriasis. Gels based on aloe vera presented anti-tumor, anti-inflammatory, anti-bacterial, anti-viral, antiseptic and wound healing properties. Due to its antibacterial, anti-inflammatory and moisturizing effects, aloe vera is recommended to treat wounds and skin burns [151]. The success of using of nanoformulated preparations depends on the correct selection of the surfactant component. Commonly used surfactants include Tween^®^ (polysorbates), Cremophor^®^ (mixture of macrogol glycerol hydroxystearate, PEG-40 castor oil, polyoxyl 40 hydrogenated castor oil), Transcutol^®^ P (diethylene glycol monoethyl ether), Plurol Oleique^®^ (polyglyceryl-3-oleate), Plurol Isostearique^®^ (isostearic acid ester of poly-glycerols and higher oligomers) and Labrasol^®^ (mixture of mono-, di- and tri-glycerides of C8 and C10 fatty acids, and mono- and di-esters of PEG). Perspective low toxicity non-ionic surfactant decyl glucoside from plant-derived feedstocks is an effective alternative to polyethoxylated sulfate containing detergents. Decyl glucoside is convenient for sensitive skin and baby products [152]. Lecithin is a natural surfactant applied in past centuries for pharmaceuticals. Lecithin is easily absorbed by epidermis, but its usage is restricted by low chemical stability [153]. Viscosity enhancing agents (e.g., Carbopol^®^, Aerosil^®^, gelatin) are offered to get the desired consistency of the product in wound [154].

### 5.3. Microemulsions in Cancer Theranostics

Due to their unique physico-chemical characteristics, microemulsions found their application in cancer diagnosis and drug delivery. Several compositions have been able to meet the demands for targeted delivery and controlled release, local concertation of drugs or their prolonged circulation, or even microenvironmental drug shielding.

First of all, the aspect of targeted anticancer delivery. Tween 80-based MEs reportedly can support droplets of ~10 nm size for drug delivery without affecting the cell viability for colon cancer [55]. Current research shows that the surfactant layer can be modified for enhanced uptake, such as in the case of [155]. Functionalizing the droplets with transferrin led to an increase in ME uptake by human lung carcinoma A549 cells. This effect was later confirmed in vivo with transferrin-ME having higher efficacy and active tumor targeting. Transferrin proved to be an affective surface modification for ovarian cancer as well [156]. Another approach for drug delivery is magnetic targeting. It is possible to achieve stable magnetite dispersion in MEs with a droplet size of >70 nm. The magnetic susceptibility of emulsions will be lower than that of magnetite and depends on phase composition; however, it is still sufficient for magnetic targeting. These magnetic microemulsions have shown great targeting potential during in vivo studies on 4T1 breast carcinoma bearing BALB/c mice, both in CT scans and histological examination [157].

The supporting case of delivery is controlled release via ME. For example, since breast cancer is the most common type for women, it is important to reach for new approaches besides the usual mastectomy and radiochemotherapy. In [158], the novel biocompatible non-aqueous microemulsion of phosphatidylcholine/tricaprylin/propylene was obtained. Nanosized droplets and chemical nature allowed for incapsulation of the lipophilic drug fenretinide. This microemulsion served as a drug depot in vitro, with slow drug release corresponding with significantly raised cytotoxicity against breast cancer cell lines at low administered concentrations. Interestingly enough, the composition also greatly reduced cell migration and spheroid viability. During in vivo experiments, ME confirmed previous results. Additionally, it did not cause any local effects and tissue damage upon administration, and was also able to retain a fluorescent marker in tumor zone. Furthermore, the authors of [159] developed a transdermal ME carrier. A classic w/o microemulsion included a plant-derived methyl-dihydrojasmonate with reported selective anticancer activity. TEM confirmed spherical ME capsules of less than 200 nm diameter. In ex vivo permeability experiments, a system with Transcutol^®^ and Labrasol^®^ (commercial non-ionic surfactants are recommended because of their low irritancy, high stability, biodegradability and low toxicity) did, in fact, prove to suit transdermal carrier characteristics. It has shown a great cytotoxic anticancer effect both in in vivo and in vitro studies. Same results can be seen for other cancer types including lung, colon and breast adenocarcinoma [60,61,62].

While drug shielding is still in development, it has already yielded promising results. The idea is that the molecule embedded in physically stable droplets is protected from early elimination. In [63], it was shown that this protection is achieved by w/o MEs with a ~100 nm diameter and negative surface charge. Incapsulated oxaliplatin was not available for elimination by glutathione pump, and thus drastically lowered cancer cell proliferation in vitro.

Supporting previous effects, MEs can also be modified for prolonged or even controlled drug circulation. In [64], a folate-linked ternary composition was proposed. This microemulsion showed high accumulation in Hep2 cells and after injection in vivo. Additionally, the association of droplets to cells could be inhibited by microconcentrations of folic acid. It has been reported that the microemulsion incapsulation adds to the biocompatibility of therapeutic agents. In [57], it has been shown that the surfactant layer of droplets lowers protein binding to drugs, which also played a role in controlled release. The formulation had the physicochemical characteristics for successful oral administration, substantially enhancing the bioavailability. These examples show how microemulsions can act as a multifunctional delivery systems, enhancing the bioavailability of the therapeutic or diagnostic agent while also protecting it from different components of biological fluids.

In some cases the drug, such as tripterine, has a small therapeutic window, leading to complications in clinical trials and application. Apparently some microemulsions allow for balancing of the toxicity and antitumor effects of such drugs. A system of tripterine modified with both transferrin and cell-penetrating peptide SA-R6H4 (Tf/SA-R6H4-TBG-MEs) for combinational and tumor-targeted cancer therapy was investigated. During the tests, the inhibitory concentration was nearly six-fold lower than that of free tripterine, and the peptide modulated the higher cellular uptake. In vivo trials showed a great antitumor effect and longer survival rate due to modification, as well as significantly lower toxicity towards liver and kidney tissues [160,161]. The combinatory treatment strategy, abbreviated as FOLFLOX, which is an assembly of three anticancer compounds, also demonstrated certain side effects due to low specificity and efficacy taken together with toxic effects and a long duration of treatment. Controlled emulsion microenvironmets provide a new strategy for enhanced blood circulation. In vivo studies in specimens with colon cancer show that significantly stronger chemo-immunotherapeutic responses were achieved by encasing FOLFLOX components in lipid nanoparticle emulsion [162]. Aptamers have also proven to be substantial in cancer targeting. Thus, Zhou et al. [163] showed that an aptamer-anchored microemulsion bearing combinatory drugs was investigated. The composition was able to release the drugs depending on pH. In both in vivo and in vitro experiments, this microemulsion showed outstanding tumor growth inhibition and lower systemic toxicity, thus implicating that controlled and tumor-targeting microemulsions loaded with combination of drugs should be investigated as a form of clinical co-delivery.

An interesting case for microemulsion compositions is the addition of well-known liposome delivery strategies. On multiple occasions, liposomes have proven to be of high bioavailability and efficacy in targeted delivery. A group in [164] reports that they have achieved sequential codelivery in anticancer therapy. They have fabricated multicomponent-based liposomes loaded with sodium tanshinone IIA sulfonate (STS) and a microemulsion of celastrol (CM). In the tumor microenvironment, pH-dependent depletion of STS from the liposomes led to the subsequent release of CM. This induced great coordinated cytotoxicity against breast cancer cells. Additionally, this delivery system exhibited low systemic cytotoxicity compared to celastrol monotherapy. These results are promising for coordinated treatment of solid tumors.

Aside from traditional biologically active compounds, ferromagnetic particles have been investigated due to local magnetic hyperthermia phenomena. This allows for their use as an alternating magnetic field to locally damage cancer cells with heat produced by ferromagnetic material. An oil-in-water microemulsion system may be used to stabilize and functionalize magnetic nanoparticles which have their limitations in theranostics due to aggregate formation and low biocompatibility. The Zn-Fe oxide covered by silica using such a microemulsion had great heating characteristics, as well as better biocompatibility and cellular intake [165].

During the past few years, cancer therapy has made great success in antitumor vaccines. This strategy usually utilizes solid nanodimensional granules as the base for administration. To assure the biocompatibility of delivery vehicles, lipid and protein complexes are implemented, but to this day, the toxicity and control of physicochemical properties is of outstanding complexity. Microemulsions here can serve both as a stabilizer and a controlled microenvironment. In [58], a reverse microemulsion was implemented as a synthesis media for pyrophosphate granules. The resulting nanovaccine core was stable and of ≈50 nm in diameter with monodispersity. The same idea was used in [59] to achieve size control of nanoparticles. The same approach with microemulsion phases bearing the core of the nanovaccine and its therapeutically relevant component allows for achieving greater biocompatibility and cellular uptake [56]. Overall, microemulsion systems are an emerging strategy for cancer nanovaccine improvement.

Apart from experimental research, there are notable clinical trials concerning microemulsions in cancer theranostics (Table 4) [166] (https://clinicaltrials.gov/, accessed on 27 March 2023).

These examples depict microemulsion compositions as an emerging drug delivery and release system. Through combining different phases and modifying the surface or chemical properties of resulting droplets, it is possible to achieve magnetically or even receptor-controlled depots for anticancer drugs.

## 6. Conclusions

According to the current experimental and theoretical results of colloid science, microemulsions are defined as unique physico-chemical structures forming thermodynamically stable isotropic fluid phase consisted from large amounts of nonpolar (oil) and polar (water or ionic solution) solvents. The structural features of microemulsion compositions are much more diverse than the simple droplet model of isolated liquid spheres. Surface interface between hydrophobic and aqueous areas is flexible due to its fluctuation nature, which give rise to significant entropy contributions to the energy of mixing. Unique physico-chemical properties of nanoformulated systems are expressed in the formation of various structures as drop-like, direct and inverse swollen micellar-like structures, Winsor liquid crystalline domains, bicontinuous mixtures and others. The summary of the micro- and nanoemulsion comparison is presented in Table 5.

The structures of microemulsions strongly depend on the chemical composition and temperature. The fundamental microemulsion property of practical importance is the low surface tension on the border of microdrops, which have a small dimension in the interval of 10-100 nm. Due to low surface tension, microemulsions can be used as effective emulsificators for producing numerous stable emulsions, creams and ointments of medical applications. Substantial amounts of bioactive substances can be included into such multicomponent mixtures, stabilized by a coexisting microemulsion phase. A specific microemulsion composition creates the conditions for the mutual presence of bactericidal agents such as nanoclusters Ag and labile immunoreactive proteins (e.g., enzymes, cytokines, hormones, etc.) in one medication without inactivation. Another perspective approach of reverse w/o microemulsion is to use microdroplets as microreactors for the fabrication of magnetic iron oxide superparamagnetic nanoparticles for MRI diagnostics, semiconductor quantum dots for confocal cell microscopy, and highly dispersed catalytically active metals for topical treatment of burns and trophic ulcers. Thus, according to present published results the multicomponent colloidal systems containing the microemulsion phase have the great potential for development of new effective delivery means for treatment and clinical diagnostics.

The overall outlook for emulsion systems in modern biomedicine is a very promising one. On the one hand, they have substantial abilities to solve or incapsulate therapeutic and diagnostic agents, combined this with their tissue permeation. On the other hand, microemulsions have a long shelf life due to their thermodynamic stability, while nanoemulsions resist degradation by being kinetically stable. Both systems have available and varying strategies of synthesis. Thus, according to present published results the multicomponent colloidal systems containing the microemulsion phase have great potential for the development of new effective delivery means for treatment and clinical diagnostics.

## Figures and Tables

**Table 1:** HLB values and solubility.

HLB	Solubility Type in Water	Emulsion Type
0–4	Non-dispersible	w/o
6	Low dispersion	
8	Unstable opaque dispersion	wetting agent
10	Stable opaque dispersion	
12	Semitransparent solution	o/w
14–16	Transparent solution	

**Table 2:** Common surfactants and their properties.

Name	Oxyethylated Groups	HLB Value	
		Observed	Theoretical
SDS	-	40	40
Potassium oleate	-	20	20
Sorbitan monooleate (Tween 80)	20	15	15.8
Sorbitan monooleate	10	13.5	12.5
Sorbitan monooleate (Tween 81)	5	10	10.9
Sorbitan monooleate	2	7	7
Sorbitan monolaurate (Span 20)	-	8.6	8.5
Sorbitan monolaurate (Span 40)	-	6.7	6.6
Sorbitan monolaurate (Span 60)	-	5.9	5.7
Sorbitan monolaurate (Span 80)	-	4.3	5.0
Propylene-glycol monolaurate	-	4.5	4.6
Propylene-glycol monostearate	-	3.4	1.8
Glycerol monostearate	-	3.8	3.7
Sorbitan tristearate	-	2.1	2.1
Sorbitan tristearate	-	1.8	-

**Table 3:** Common applications for microemulsion compositions in biomedicine.

Nanoformulation	Type of Formulation	Chemical Composition	Application	Active Agent	Mechanism of Action	Advantage	Ref.
Nanocarriers of pharmaceuticals *	O/W Reverse micelles	Oil/Polyoxyethylated hydrogenated oils/H_2_O Oil/Polyoxyethylated sorbitan/H_2_O	Cancer Diabetes	Anti-inflammatory drugs Cytokines Hormone (insulin) Immunomodulatory proteins Doxorubicin	Nanocarrier Receptor recognition Endocytosis Ag-Ab binding	Enhancement of bioavailability Targeted delivery Sustained and controlled drug release Promotion of intestinal absorption	[54,55,56]
Diagnostics *	Multiple emulsion Nanosuspensions	NPs, MNPs in micellar coat	MRI, Cri, US Laser Radiology	Fe, Gd, Au, mNPs, Qdots	Nanoscale Noninvasive Low toxicity	High sensitivity Differential body distribution	[57]
Nanovaccines *	w/o/w o/w	Chitosan Gyaluronan Nonionic surfactants	Influenza Cancer Viral infections Hepatitis	DNA, siRNA Inhibited enveloped virus Virus capsular protein	Adjuvant action	Efficient oral antigen deliver Nasal, Respirative efficiency Nucleic acid transfection	[58,59]
Microcapsulation Nanocapsules Nanospheres	Microemulsion method Double emulsion Ionotropic gelation Coacervation	Mineral oil/nonionic surfactant/cosurfactant{salts of aliphatic acids) Polylactide, chitosan, gelatin	Versatile disorders Chronic diseases Enzyme protection	Non-steroidal anti-inflammatory drugs Protein Antibiotic	Chemical stability Prescribed release	Bioavailability Sustained delivery Prolonged action	[60,61,62,63,64]
Antimicrobial agents Antivirus drugs	Nanoemulsion Multiple emulsion. Lotions	Antibiotics from all major groups	Tuberculosis Gram-negative, Gram-positive pathogens. Acute and chronic wounds, trophic ulcers	Antibiotics, Ag Antiseptic chemicals (H_2_O_2_, J2, sodium hypochlorite, Chlorhexidine, detergents)	High penetration to infected area	Enhanced action	
Cosmetic care products	Emulsions Creams Gels Patches	Gyaluronan, collagen, gelatin Emulgators Mineral and vegetable oil		Vitamins Amino acids Pigments		Anti-age action High skin hydration	
Bioactive nutrients and supplements	o/w/o	Vegetable oil, water Natural emulgators, wax	Diet and health Liver, gastrointestinal health, inflammatory bowel,	Lipids, amino acids, Polyphenols, alcoloids Omega-6,3 polyunsaturated Fatty acids Minerals (Se, Mg, Zn, Mo)	Overcome the barriers of mucosal surface in gastrointestinal tract	Facilitated absorption and assimilation	

* including cancer theranostics

**Table 4:** Clinical trials (completed or active) including microemulsions applications.

Trial Identifier	Emulsion Features	Drug	Target Cancer	Country	Phase
NCT00664170	Inverse, injectable	Docetaxel	Breast, lung, prostate	USA	1
NCT00034151	Injectable	Paclitaxel	Ovarian	USA	2
NCT02367547	Nanoemulsion	Aminolevulinic acid	Basal cell carcinoma	Finland	1, 2
NCT03311334	Controlled release	DSP-7888 ^1^	Melanoma, liver, colorectal	USA	1, 2
NCT02498665	Controlled release	DSP-7888 ^1^	Glioblastoma, ovarian, pancreatic	USA	1
NCT00385177	Injectable	SN2310 ^2^	Breast, pancreatic, colorectal	USA	1
NCT00432562	Injectable	Vinorelbine Tartrate	Breast, lung	Argentina	1
NCT00034164	Injectable	S-8184	Lung	Russia	2

^1^ Adegramotide, experimental peptide vaccine; ^2^ experimental lypophilic drugs.

**Table 5:** Micro- and nanoemulsion comparison.

Characteristic	Microemulsion	Nanoemulsion
Thermodynamic stability	+	–
Kinetic stability	+	+
Droplet size, nm	200–1000	<200
Spontaneous forming	+	−
Dilution-dependent morphology	+	−
Order-specific formulation	−	+

## Data Availability

Not applicable.
